# βIII‐tubulin suppression enhances the activity of Amuvatinib to inhibit cell proliferation in c‐Met positive non‐small cell lung cancer cells

**DOI:** 10.1002/cam4.5128

**Published:** 2022-08-10

**Authors:** Simon Brayford, Alastair Duly, Wee Siang Teo, Tanya Dwarte, Estrella Gonzales‐Aloy, Zerong Ma, Laura McVeigh, Timothy W. Failes, Greg M. Arndt, Joshua A. McCarroll, Maria Kavallaris

**Affiliations:** ^1^ Children's Cancer Institute Lowy Cancer Research Centre, UNSW Sydney Australia; ^2^ Australian Centre for NanoMedicine, UNSW Sydney Australia; ^3^ School of Clinical Medicine UNSW Medicine and Health Sydney Australia; ^4^ ACRF Drug Discovery Centre for Childhood Cancer Children's Cancer Institute, Lowy Cancer Research Centre, UNSW Sydney Australia

**Keywords:** Amuvatinib, cancer therapeutics, ERK Signalling, MP‐470, non‐small cell lung cancer, βIII‐tubulin

## Abstract

Non‐Small Cell Lung Carcinoma (NSCLC) remains a leading cause of cancer death. Resistance to therapy is a significant problem, highlighting the need to find new ways of sensitising tumour cells to therapeutic agents. βIII‐tubulin is associated with aggressive tumours and chemotherapy resistance in a range of cancers including NSCLC. βIII‐tubulin expression has been shown to impact kinase signalling in NSCLC cells. Here, we sought to exploit this interaction by identifying co‐activity between βIII‐tubulin suppression and small‐molecule kinase inhibitors. To achieve this, a forced‐genetics approach combined with a high‐throughput drug screen was used. We show that activity of the multi‐kinase inhibitor Amuvatinib (MP‐470) is enhanced by βIII‐tubulin suppression in independent NSCLC cell lines. We also show that this compound significantly inhibits cell proliferation among βIII‐tubulin knockdown cells expressing the receptor tyrosine kinase c‐Met. Together, our results highlight that βIII‐tubulin suppression combined with targeting specific receptor tyrosine kinases may represent a novel therapeutic approach for otherwise difficult‐to‐treat lung carcinomas.

## Graphical abstract

Amuvatinib (MP‐470) inhibits lung cancer cell proliferation following βIII‐tubulin suppression as demonstrated by decreased Ki67 and BrdU‐incorporation using ELISA. These data show that suppressing the survival factor βIII‐tubulin, the activity of Amuvatinib (MP‐470) is enhanced.

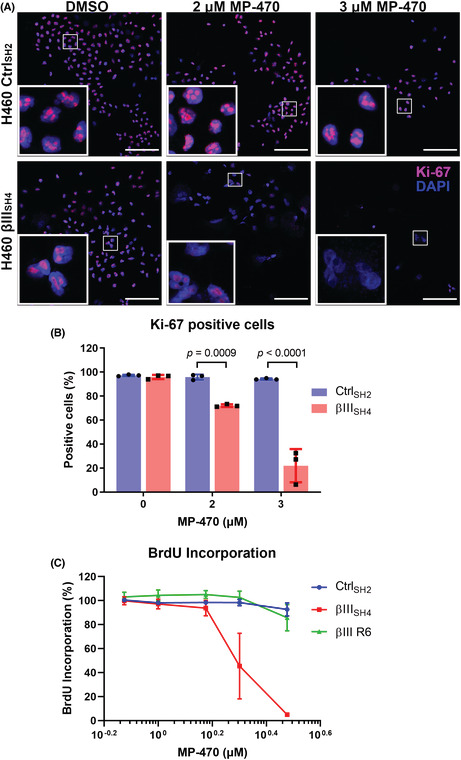

## 1 INTRODUCTION

Lung cancer is one of the most common cancer types and remains a leading cause of cancer‐related mortality worldwide.^1^ The most common sub‐type of lung cancer, non‐small cell lung carcinoma (NSCLC), represents more than 80% of cases and has a dismal 5‐year survival rate of less than 15%.^2^ Chemotherapy remains the most effective treatment option however problems with drug resistance and toxicity present major clinical challenges.^3^

Microtubules form a major component of the cell cytoskeleton and alterations in the expression of microtubule proteins are increasingly correlated with more aggressive and treatment‐refractory cancers.^3^ Intracellular signalling requires trafficking of proteins throughout the cell via the microtubule network, thus modulation of microtubule proteins has an expected impact on cell signalling in tumour cells.^4^ In addition to general effects on signal transduction, microtubules have been shown to regulate specific signalling pathways, including the extracellular regulated kinase (ERK) pathway which is involved in mediating a range of cellular activities, including cell proliferation.^5^

There is growing evidence for tubulin isotypes differentially influencing microtubule behaviour and more broadly regulating cancer biology and chemotherapy resistance.^6^ One such isotype, βIII‐tubulin, is normally expressed in neurons, but is aberrantly expressed in NSCLC and other epithelial cancers,^3^, ^7^ where its expression is associated with poor patient outcome and chemotherapy resistance.^8^, ^9^, ^10^, ^11^ Recent evidence suggests that βIII‐tubulin influences microtubule dynamics through interactions with its carboxy‐terminal tail,^12^ and several studies have shown that βIII‐tubulin may serve as a downstream target of several known oncogenic pathways.^13^, ^14^

Previously, our group has demonstrated that βIII‐tubulin impacts the PI3K/AKT cell survival pathway in NSCLC cells,^15^ highlighting an association between this tubulin isotype and kinase signalling. Kinase signalling pathways have been shown to drive many of the hallmark phenotypes of tumour biology^16^ and as such, kinases are increasingly being explored as targets for inhibitors.^17^ Inhibition of distinct kinase signalling pathways can be less cytotoxic to non‐cancerous cells than conventional chemotherapy,^18^ and kinase inhibitors are now prescribed for a wide range of malignancies.^19^, ^20^ For example, Dong et al recently reported that the FDA approved HER2 inhibitor Mubritinib alone or in combination with cisplatin could significantly inhibit NSCLC growth in vitro and in vivo.^21^ Problems with drug resistance and compromised efficacy however continue to present critical challenges in clinical and experimental oncology,^22^, ^23^ highlighting a need to find alternative ways to sensitise tumour cells to such agents.

Given the links between βIII‐tubulin and kinase signalling, and that βIII‐tubulin suppression has been shown to increase sensitivity of NSCLC cells to chemotherapy,^11^ we investigated whether suppression of βIII‐tubulin might sensitise NSCLC cells to small‐molecule kinase inhibitors. Using our unique βIII‐tubulin gene knockdown and rescue cell models in combination with a high‐throughput kinase inhibitor screen, we identified that activity of the multi‐targeted kinase inhibitor Amuvatinib (MP‐470) was enhanced by βIII‐tubulin suppression. We show that MP‐470 combined with βIII‐tubulin suppression significantly reduced cell proliferation in both 2D cultures and 3D tumour spheroids. Finally, we demonstrate that this effect is mediated through altered ERK signalling downstream of the receptor tyrosine kinase c‐Met, and that RNAi mediated knockdown of c‐Met expression reverses the sensitivity phenotype. Together, our results highlight that Amuvatinib (MP‐470) combined with βIII‐tubulin inhibition may offer a novel therapeutic strategy to arrest tumour growth in c‐Met‐positive non‐small cell lung cancer.

## 2 MATERIALS AND METHODS

### 2.1 Cell culture

βIII‐tubulin shRNA expressing cells (H460 βIII_SH4,59_, and A549 βIII_SH61_), stable control shRNA expressing cells (H460 βIII_SH1,2_, and A549 βIII_SH27_) and H460 βIII‐tubulin “rescue” cells (H460 βIII R6) were all developed and characterised as previously described.^11^ The use of all genetically modified cell lines was done in accordance with the UNSW GMO notification system under Exempt Dealing 21–22. H1299 NSCLC cells were obtained from ATCC (ATCC, cat# CRL‐5803). All cell lines were validated by STR profiling at the Garvan Institute and were routinely tested and found to be free from mycoplasma contamination. Cells were maintained in RPMI‐1640 medium (Gibco, cat# A1049101), supplemented with 10% FBS, at 37°C and 5% CO_2_. For routine maintenance, cells were cultured in T75 plastic cell culture flasks (Corning, cat# 430641U) and split via trypsinisation approximately every 3 days or when approaching confluence.

### 2.2 Kinase inhibitor screens

High‐throughput, cell based screens were performed at the ACRF Drug Discovery Centre for Childhood Cancer, Children's Cancer Institute, Lowy Cancer Research Centre, UNSW Sydney, Australia. The primary screen consisted of a total of 210 kinase inhibitors from a WEHI kinase inhibitor library (Walter and Eliza Hall Institute, Melbourne, Australia) tested at single, fixed concentrations (5 μM). Briefly, 125 cells per well were seeded in 384‐well cell culture plates (Thermo Fisher, cat# 464718) for 24 h prior to drug exposure for 72 h and cell viability was determined using Alamar blue assay. Compounds were ranked by difference in cell viability, and top 10 were selected for secondary screen. Secondary screen was performed using conditions described above in 5‐point dose–response (0.625–10 μM) in triplicate.

### 2.3 Alamar blue cell viability assay

Alamar Blue assay was used to assess cell viability. Briefly, 1000 cells per well were seeded in 96‐well cell culture plates (Greiner Bio One) for 24 h prior to drug exposure for 72 h. Alamar Blue was then added to each well to a final concentration of 10% (v/v) solution of Alamar Blue dye. A cell‐free well was included as a blank. Following 4 h incubation, absorbance was measured at 570 nm with a 600 nm reference filter using a Benchmark Plus Microplate reader (BioRad). The results were averaged over 3 different independent experiments with triplicate wells measured per experiment.

### 2.4 Clonogenic assay

Clonogenic assays were performed as previously described.^7^ Briefly, 120 NSCLC cells per well were added to six‐well plates (Greiner Bio One), and allowed to attach for 24 h. MP‐470 (Selleckchem, cat# S1244) was then added at various concentrations (0–3 μM) in 2 ml of medium for 72 h, after which, drug‐containing medium was replaced with normal growth medium. Plates were examined daily for formation of visible colonies (defined as being comprised of >50 cells, as counted by eye using a standard tissue‐culture microscope). Fixing and staining was then performed simultaneously by added 1 ml per well of 0.5% crystal violet in methanol for 1 h at room temperature. Wells were rinsed multiple times with milli‐Q water, before being imaged using a GelDoc EZ imager (BioRad) and individual stained colonies manually counted. Surviving fraction was calculated using the formula: colony number/(number of cells seeded × plating efficiency), where plating efficiency = colony number divided by number of cells seeded in drug‐free wells. The results were averaged over 3 different independent experiments.

### 2.5 Immunofluorescence and confocal microscopy

Cells were seeded in Lab‐Tek glass chamber slides (Nunc, Roskilde, Denmark) at 1 × 10^3^ cells per cm^2^ and allowed to adhere 24 h prior to treatment with either MP‐470 or DMSO (Sigma Aldrich, cat# D2650) for a further 72 h. Cells were then fixed with paraformaldehyde (4% [w/v] in PBS) for 20 min at room temperature, permeabilised with 0.5% Triton X‐100 (Sigma Aldrich, cat# 11332481001) in PBS for 15 min at room temperature, blocked with 5% FBS in PBS for 30 min at room temperature with brief PBS washes between steps. Cells were stained for Ki‐67 (Cell Signalling Technology, cat# 9449; diluted 1 in 500 in 2% FBS in PBS) for 1 h at room temperature. Cells were thoroughly rinsed with PBS before secondary antibody staining with AlexaFluor® 568‐conjugated goat anti‐mouse antibody (Thermo Fisher, cat# A‐11031; diluted 1 in 500 in 2% FBS in PBS) for 1 h at room temperature. Following final thorough rinsing with PBS, coverslips were mounted using Prolong Gold mounting medium containing DAPI (Thermo Fisher, cat# P36931) to visualise nuclear material. Images were then acquired using Zen Microscope Software (Zeiss, Oberkochen, Germany, Black edition V.2.1,) controlling an LSM 780 inverted confocal microscope (Zeiss) using filter sets for 405 nm or 561 nm lasers with a 20X (NA 0.8) Plan‐Apochromat air‐immersion objective lens (Zeiss). The results were averaged over 3 different independent experiments.

### 2.6 BrdU incorporation assay

Cell proliferation was determined by measuring the incorporation of bromodeoxyuridine (BrdU) into DNA using the Colorimetric BrdU ELISA proliferation assay kit (Roche, cat# 11647229001). And 1000 cells per well were seeded in 96‐well cell culture plates (Greiner Bio One) for 24 h prior to MP‐470 exposure for 72 h. BrdU labelling reagent (supplied with kit) was diluted 1:100 with sterile culture medium (resulting concentration: 100 μM BrdU) and 100 μl added per well for 5 h before fixation/denaturation was performed using the fix/denat regent supplied with the kit. BrdU incorporation assay was then carried out as per manufacturer's instructions. The results were averaged over at least 3 different independent experiments with 4 wells measured per experiment.

### 2.7 Live/Dead staining assay

To assess Live/Dead cells, a multiplex viability/cytotoxicity kit for mammalian cells (Thermo Fisher, cat# L3224) was used. And 1000 cells per well were seeded in 96‐well cell culture plates (Greiner) for 24 h prior to MP‐470 exposure for 72 h prior to the assay, EthD‐1 (included in kit) was diluted in PBS to a final concentration of 4 μM. Calcein AM (included in kit) was then added to the diluted EthD‐1 solution to a final concentration of 2 μM, culture medium was then removed and 100 μl of the staining solution was added per well and incubated 45 mins at Room temperature. Fluorescence was then read at both 645 nm (dead cells) and 530 nm (live cells) for all wells using a Synergy Neo2 Multi‐Mode Microplate Reader (BioTek). Calculations were performed according to kit manufacturer's instructions to calculate live and dead cell numbers. The results were averaged over 3 different independent experiments with 4 wells measured per experiment.

### 2.8 Caspase‐9 activity assay

To assess apoptosis, 1000 cells /well were seeded in 96‐well cell culture plates (Greiner) for 24 h prior to MP‐470 exposure for 72 h prior to the assay, a Caspase‐9 assay kit (Abcam, cat# ab65607) was then used according to manufacturer's instructions. Fluorescence was then read at 400 nm excitation/505 nm emission using a Synergy Neo2 Multi‐Mode Microplate Reader (BioTek). And 5 μM Staurosporine was used a positive control for apoptosis. The results were averaged over three different independent experiments with triplicate wells measured per experiment.

### 2.9 Cellular senescence staining

Senescence detection was performed using an assay kit (Abcam, cat# ab65351) according to manufacturer's instructions. Briefly, following drug treatments in 6‐well plates (100 μM H_2_0_2_ was used as a positive control for senescence), cells were rinsed once with PBS, then fixed with Fixative Solution (supplied with kit) for 15 min at room temperature. Cells were then washed twice with PBS before the addition of staining solution (X‐Gal, supplied with kit) overnight at 37°C in the dark. Cells were then analysed using a brightfield, upright microscope (Olympus, Shinjuku, Japan, BX3M) to observe and record development of blue colour to indicate cellular senescence (20× magnification).

### 2.10 In vitro cell cycle analysis

Cells 100,000 per well were seeded in 6‐well plates for 24 h before treating with either 3 μM MP‐470 or DMSO for 48 h. Adherent and cells in suspension were then collected and counted. And 4–8 × 10^5^ cells per condition were fixed in 70% ethanol at −20°C for 24 h. Fixed cells were vortexed briefly and washed twice with ice cold PBS with centrifugation at 250×g for 15 min at 4°C in between washes, prior to resuspending in staining solution (50 μg/ml propidium iodide (Sigma‐Aldrich, Cat# P4864), 2 μg/ml RNase, Dnase‐free (Roche, Cat# 11119915001) and 0.6% Triton X‐100). Following incubation on ice for 20 min in the dark, cells were centrifuged at 250×g for 15 min at 4°C and resuspended in ice cold PBS. Stained cells were subsequently analysed using a BD LSRFortessa™ SORP X‐20 (UNSW Mark Wainwright Analytical Centre) at YG585‐A acquisition wavelength. Histograms were generated using FlowJo v10.7.1 (FlowJo) and cell cycle analysis performed using the Watson Pragmatic model for fitting Gaussian curves to each cell cycle phase.

### 2.11 3D tumour spheroids

3D multicellular spheroids were generated as previously described.^24^ Briefly, cells were seeded in 96‐well Clear Round Bottom Ultra‐Low Attachment Microplates (Corning, cat# 7007) and monitored daily. Seeding densities were optimised for H460 cells so that tumour spheroids fell within a size range of approximately 300–500 μm, and were of consistent macroscopic morphology (aspect ratio) across wells on day 4. Spheroids with an average diameter ~ 300–500 μM after 4 days growth were treated with either DMSO or 0–5 μM MP‐470 and imaging commenced. Images were captured every 2 h using the 4x objective (NA 0.2) of an IncuCyte S3 widefield inverted live cell system (Essen BioScience) housed inside a cell culture incubator (37°C, 5% CO_2_). Spheroid size (area) was measured using ImageJ software (NIH, V.1.53c) and calculated as fold‐change from time = 0 h to account for any initial differences in spheroid size.

### 2.12 Gene expression analysis

The expression of MP‐470 target genes in H460 and A594 cells was examined using Reverse Transcription quantitative PCR (RT‐qPCR). Total RNA was extracted from cell using the Qiagen Rneasy Mini kit (Qiagen, cat# 74014) according to the manufacturer's instructions, and Reverse‐Transcription PCR performed using 1 μg of extracted RNA and the Applied Biosystems High‐Capacity cDNA Reverse Transcription kit according to the manufacturer's instructions in 10 μl reactions using random hexamers. Gel‐based PCR was then performed using an AmpliTaq Gold DNA polymerase, LD kit (Applied Biosystems, Waltham MA, USA, cat# 4338856). Briefly, PCR reaction mixtures were set up according to the manufacturer's instructions, using 10 ng of cDNA as template using the following cycling conditions: 95°C for 10 min, followed by 45 cycles of 95°C for 15 s, 65°C for 15 s which dropped 0.2°C each cycle, 72°C for 15 s, and then 40°C for 30 s for cooling. PCR products were run on a 2% agarose gel with a Quick‐Load 100 bp DNA Ladder (New England Biolabs, cat# N0467S) to confirm gene expression. Primer pairs for MP‐470 targets were designed using NCBI Primer‐BLAST program and designed with the following criteria: amplicon size of between 100–150 bp; amplicon contained at least one primer spanning an exon‐exon junction, and additionally separated by at least 1 intron top exclude genomic DNA contamination. Amplicons were then validated via Sanger Sequencing by the Ramaciotti Centre for Genomics, UNSW Sydney, Australia. Primers were ordered from Intergraded DNA Technologies (sequences shown in Table S1). β2‐microglobulin was used as a reference gene and was amplified from the same cDNA in parallel as a loading control (β2‐Microglobulin QuantiTect Primer Assay, Qiagen, cat# qt00088935). RNA sourced from THP1 cells were used as a positive control for *FLT3*, *KIT* and *RET* expression.

### 2.13 Western blotting

Total cellular protein was extracted by first rinsing cells with cold PBS and then scraping with RIPA buffer (50 mM Tris HCl, 150 mM NaCl, 1.0% (v/v) NP‐40, 0.5% (w/v) Sodium Deoxycholate, 1.0 mM EDTA, 0.1% (w/v) SDS and 0.01% (w/v) sodium azide at a pH of 7.4) in the presence of protease and phosphatase inhibitors (Roche, cat# 11697498001 & 4906845001), and incubating on ice for 40 mins for lysis to occur. Lysates were then centrifuged at ~13,000×g for 20 min and supernatants transferred to fresh tubes. To ensure equal protein loading onto gels, protein concentration was determined using a BCA assay kit (Pierce, cat# 23225) as per the manufacturer's instructions. And 20 μg of total cellular protein was then loaded per well and resolved on 10% SDS‐PAGE gels before wet electro‐transfer (2 h at 200 mA) onto Immobilon‐P PVDF membranes (pore size 0.45 μM; Merck Millipore, cat# IPVH00010). Following 30 mins blocking in 5% (w/v) Bovine Serum Albumin (Sigma Aldrich, cat# A9418) in TBST, immunoblotting was performed overnight at 4°C using primary antibodies (all 1 in 1000 dilution in 2% BSA in TBST unless otherwise stated) against: Axl (Cell Signalling Technology [CST] cat# 8661), c‐Met (CST, cat# 8198), PDGFRα (CST, cat# 3174), PDGFRβ (CST, #3169), PTEN (CST, cat# 9559), MEK1/2 (CST, cat# 9126), phospho‐MEK1/2 (Ser217/221) (CST, cat# 9154), ERK (CST, cat# 4695) or phospho‐ERK1/2 (Thr202/Tyr204) (CST, cat# 4376). Membranes were then washed thrice with TBST and incubated with species‐appropriate HRP‐conjugated anti‐IgG secondary antibodies (Dako, cat# P044701‐2 or # P044801‐2) at 1 in 10,000 dilution in 2% BSA in TBST for 1 h at room temperature. Following a final wash step, chemiluminescence was performed by applying ECL western blotting substrate (Pierce, cat# 32106) according to the manufacturer's instructions. Following imaging, membranes were incubated in 2% (w/v) Bovine Serum Albumin (Sigma Aldrich) in TBST with 1 mM Sodium Azide (Amresco, cat# 6296129) to deactivate HRP‐conjugated anti‐IgG secondary antibodies, and membranes then immunoblotted as above using GAPDH (1 in 50,000 dilution; Abcam, cat# ab8245) for 1 h at room temperature. In the case of higher molecular weight targets (Axl, c‐Met, PDGFRα or PDGFRβ), membranes were cut at either 75 or 50 kDa prior to immunoblotting with primary antibodies to ensure signal acquisition was not compromised by stripping and reprobing the membranes. Lower parts of membranes were either exclusively used for GAPDH or immunoblotted for another target followed by GAPDH as mentioned above. Bands were then visualised using a GelDoc EZ imager (BioRad). Densitometric analysis of Western Blot images was performed using ImageJ software (NIH, V.1.53c). Bands were normalised to GAPDH loading control. The results were averaged over 3 different independent experiments, and presented as either relative abundance or fold change (in the case of knockdown validation).

### 2.14 Gene silencing by small interfering RNA (siRNA)

For transient gene‐knockdown studies, cells were plated onto 6‐well plates at 1 × 10^5^ cells/well and allowed to attach for 24 h. Cells were transfected with pre‐validated siRNA to knockdown human *PTEN* (Dharmacon, cat# L‐003023‐00‐0010), human *AXL* (CST, cat# 6263), or human *MET* (CST, cat# 6618) expression, or with custom designed ON‐TARGETplus siRNA (Dharmacon) against *TUBB3* (Sense strand 5′‐GAAGGAGUGUGAAAACUGCUU‐3′; Antisense strand 5′‐PGCAGUUUUCACACUCCUUCUU‐3′)^25^ or GFP (Sense strand 5′‐GCAAGCUGACCCUGAAGUUCUU‐3′; Antisense strand 5′‐GAACUUCAGGGUCAGCUUGCUU‐3′).^26^ As cell lines used have no GFP, siRNA against GFP was used as a negative control. Cells were transfected for a total of 72 h using Lipofectamine RNAiMAX reagent complexed to the siRNAs (Thermo fisher, cat# 13778100) according to the manufacturer's instructions.

### 2.15 Statistical analyses

Graphing, log‐transformations of drug concentrations, and statistical analyses were conducted using GraphPad Prism version 9.2.0 (GraphPad Software). Statistical analyses are described in each figure legend. Where a nonlinear regression analysis or a 2‐way ANOVA is used, the F distribution and the degrees of freedom are also described in the format “F(DFn, DFd) = x”, where DFn and DFd are the degrees of freedom (numerator and denominator respectively), and x = F distribution.

## 3 RESULTS

### 3.1 High‐throughput drug screen reveals kinase inhibitors that are enhanced by βIII‐tubulin suppression

**FIGURE 1:**
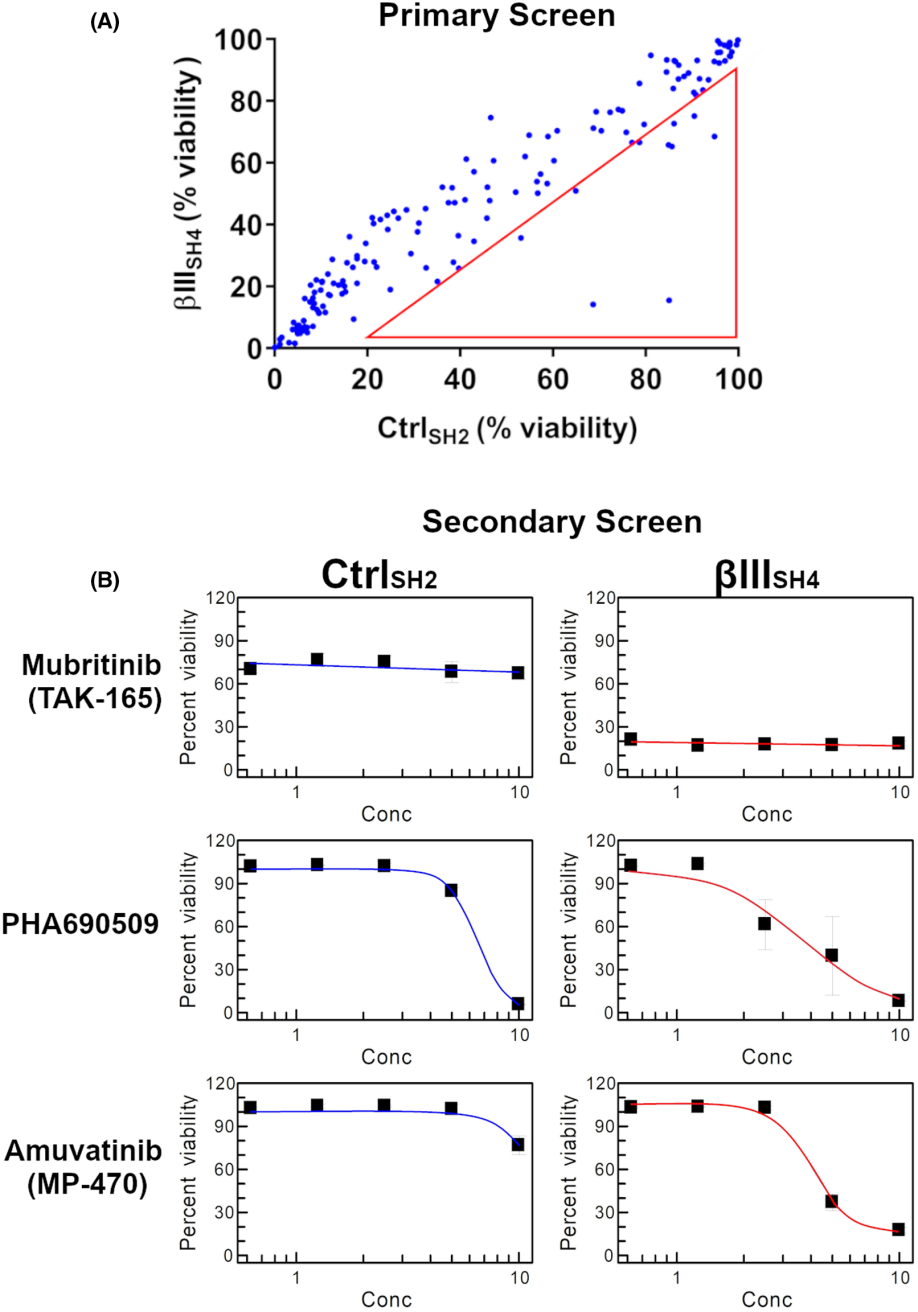
High‐throughput drug screen reveals kinase inhibitors that are enhanced by βIII‐tubulin suppression. (A) Primary Screen of 210 Kinase Inhibitors at 5 μM. Viability of βIII‐tubulin knockdown (βIII_SH4_) and Control (Ctrl_SH2_) H460 cells was determined by Alamar blue assay following 72 h exposure. Values within the red triangle were considered the top 10 compounds that were then subjected to secondary screen. (B) Secondary screen using 5‐point dose–response (0.625–10 μM) for Amuvatinib (MP‐470), Mubritinib (TAK‐165), and PHA690509. Viability of βIII‐tubulin knockdown (βIII_SH4_) and Control (Ctrl_SH2_) H460 cells was determined by Alamar blue assay following 72 h exposure.

Given our previous data showing that βIII‐tubulin levels impact kinase signalling in NSCLC cells,^15^ we investigated the ability of βIII‐tubulin suppression to enhance the anti‐tumour activity of small‐molecule kinase inhibitors. For this, we used H460 cells that had been previously established^11^ to stably express either non‐targeted shRNA (H460 Ctrl_SH2_), or shRNA targeted to silence the expression of human βIII‐tubulin (H460 βIII_SH4_). We performed a primary screen using a library of 210 kinase inhibitors at fixed concentrations and measured the % viability of H460 Ctrl_SH2_ and H460 βIII_SH4_ cells (Figure 1A). From these results, we ranked the top 10 candidates (Table 1) for further testing in a 5‐point dose–response secondary screen (Table 2). Using the IC_50_ values from this secondary screen, we identified several compounds that showed enhanced activity in H460 βIII_SH4_ cells compared to H460 Ctrl_SH2_ cells. Among these were the HER2 inhibitor Mubritinib (TAK‐165), the CDK inhibitor PHA690509, and the multi‐targeted kinase inhibitor Amuvatinib (MP‐470) (Figure 1B). While TAK‐165 and PHA690509 also specifically reduced H460 βIII_SH4_ cell viability, TAK‐165 did not produce an IC_50_ value within the concentration range tested (0.625–10 μM), and for PHA690509, the difference in IC_50_ between Ctrl_SH2_ and βIII_SH4_ cells was marginal. Amuvatinib (MP‐470) on the other hand, produced a clear IC_50_ in H460 βIII_SH4_ cells, but not in H460 Ctrl_SH2_ cells, indicating that knockdown of βIII‐tubulin sensitised H460 cells to Amuvatinib (MP‐470 hereafter). In addition, MP‐470 has previously been explored by others as part of combination therapy in platinum‐refractory small‐cell lung cancer, whereby the authors suggested further evaluation of MP‐470 in patients with high c‐Kit expression.^27^ For these reasons, we selected MP‐470 for further evaluation in our cell models of NSCLC.

**TABLE 1:**
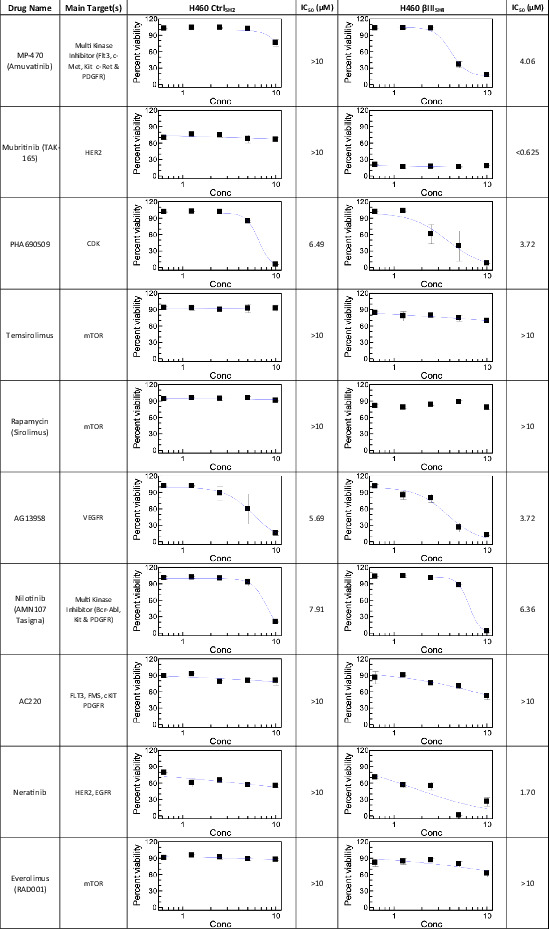
Top10 kinase inhibitors from the primary drug screen, ranked by % Viability ratio (Ctrl_SH2_: βIII_SH4_)

**TABLE 2:** Secondary screen of the top 10 compounds from primary screen as dose response (0.625 ‐ 10uM). Viability of βIII‐tubulin knockdown (βIII_SH4_) and Control (Ctrl_SH2_) H460 cells was determined by Alamar blue assay following 72 h exposure

Inhibitor	Target(s)	% Viability (Ctrl_SH2_)	% Viability (βIII_SH4_)	Viability Ratio (Ctrl_SH2_/βIII_SH4_)	Rank
Amuvatinib (MP‐470)	Flt3, c‐Met, c‐Kit, c‐Ret, axl, PDGFRα/β	85.05	15.56	5.47	1
Mubritinib (TAK‐165)	HER2	68.65	14.19	4.84	2
Nilotinib (AMN‐107)	Bcr‐abl, c‐Kit, PDGFR	94.83	68.51	1.38	3
Temsirolimus	mTOR	85.65	65.33	1.31	4
Quizartinib (AC220)	Flt3	84.90	65.77	1.29	5
AG‐13958	VEGFR	53.10	35.72	1.49	6
Rapamycin (Sirolimus)	mTOR	90.45	75.11	1.20	7
Neratinib (HKI‐272)	HER2, EGFR	39.69	25.89	1.53	8
Everolimus (RAD001)	mTOR	78.65	66.63	1.18	9
PHA690509	CDK	17.02	9.48	1.80	10

### 3.2 βIII‐tubulin suppression sensitises NSCLC cells to MP‐470

**FIGURE 2:**
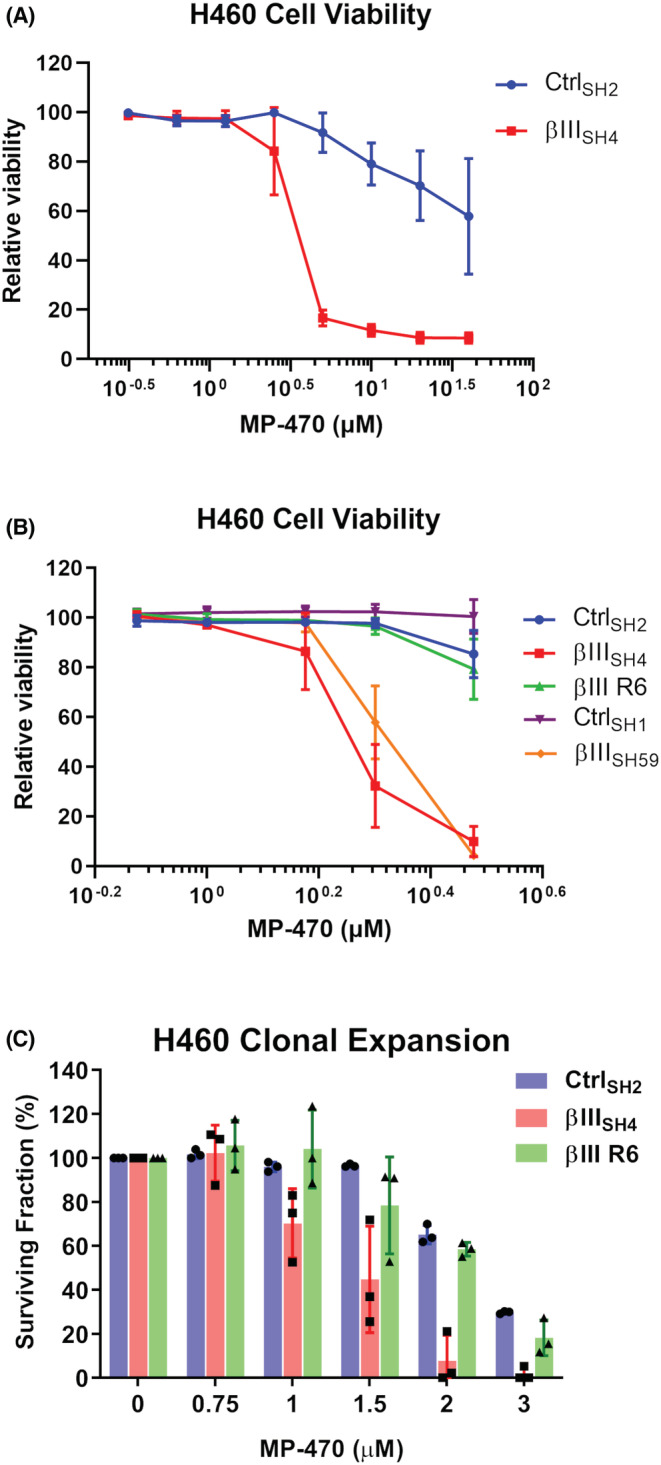
βIII‐tubulin suppression sensitises NSCLC cells to MP‐470. (A) Relative viability of Control (Ctrl_SH2_) and βIII‐tubulin knockdown (βIII_SH4_) H460 cells following exposure to MP‐470 (*n* = 3, mean ± SD; MP‐470 concentration was log‐transformed; comparison via nonlinear regression analysis, Ctrl_SH2_ vs. βIII_SH4_ F (2, 44) = 172.4, *p* < 0.0001). (B) Relative viability of Control (Ctrl_SH1_ and Ctrl_SH2_), βIII‐tubulin knockdown (βIII_SH4_ and βIII_SH59_), and βIII‐tubulin knockdown‐rescue (βIII R6) H460 cell clones following exposure to MP‐470. (*n* = 3, mean ± SD; MP‐470 concentration was log‐transformed; comparison via nonlinear regression analysis, Ctrl_SH2_ vs. βIII_SH4_ F (2, 36) = 160.6, *p* < 0.0001, Ctrl_SH1_ vs. βIII_SH59_ F (2, 26) = 322.7, *p* < 0.0001, Ctrl_SH2_ vs. βIII R6 F (2, 36) = 1.593, *p* = 0.2174, βIII_SH4_ vs. βIII R6 F (2, 36) = 135.2, *p* < 0.0001). (C) Clonogenic growth assay of Control (Ctrl_SH2_), βIII‐tubulin knockdown (βIII_SH4_), and βIII‐tubulin‐knockdown rescue (βIII R6) H460 cells following exposure to MP‐470 (*n* = 3, mean ± SD each point represents one biological replicate; comparison via nonlinear regression analysis, Ctrl_SH2_ vs. βIII_SH4_ F (2, 32) = 51.89, *p* < 0.0001, Ctrl_SH2_ vs. βIII R6 F (2, 32) = 2.751, *p* = 0.0790, βIII_SH4_ vs. βIII R6 F (2, 32) = 20.66, *p* < 0.0001).

To further validate the findings from our drug screen we performed independent Alamar Blue assays using a wider range of concentrations of MP‐470 and confirmed that H460 cells were significantly more sensitive to MP‐470 following βIII‐tubulin suppression compared to control cells (Figure 2A). To demonstrate that this result was not specific to H460 cells, we tested several other NSCLC cell lines. Firstly, we tested A549 cells expressing control (A549 Ctrl_SH27_) or βIII‐tubulin shRNA (A549 βIII_SH61_) and found that A549 βIII_SH61_ cells were significantly more sensitive to MP‐470 compared to A549 Ctrl_SH27_ cells (Figure S1A). Next, as both H460 and A549 cells are of a KRAS‐driven oncogenic background, we investigated a cell line which features the well‐characterised oncogenic driver of a p53 mutation (H1299 cells, which feature a homozygous partial deletion of *TP53* ^*28*^). As this was a parental cell line, we used siRNA to transiently knockdown expression of βIII‐tubulin (as previously described^7^), and subsequently treated these cells with MP‐470. Consistent with H460 and A549 cells with stable knockdown of βIII‐tubulin, siRNA knockdown of βIII‐tubulin significantly sensitised H1299 cells to MP‐470 compared to control cells (Figure S1B).

To determine the lowest concentration at which MP‐470 specifically reduced viability of H460 βIII_SH4_ cells, we tested a narrower concentration range and found MP‐470 to have an IC_50_ value of approximately 2 μM in these cells. In contrast, H460 Ctrl_SH2_ cells had no loss in viability at this concentration (Figure 2B Ctrl_SH2_ vs. βIII_SH4_). To confirm that this effect was not unique to just one set of shRNA‐expressing cell clones, we performed further analysis using an independent set of control and βIII‐tubulin shRNA expressing H460 cell clones and were able to replicate the results (Figure 2B Ctrl_SH1_ vs. βIII_SH59_). To further strengthen these findings and demonstrate that sensitisation of H460 cells to MP‐470 was indeed due to βIII‐tubulin protein levels, we also tested a βIII‐tubulin knockdown/rescue cell model (H460 βIII R6, described in^11^) and found that ectopic re‐expression of βIII‐tubulin protein restored βIII_SH4_ cell viability to that of the control cells (Figure 2B βIII R6). Together, these results strongly support a role for βIII‐tubulin in NSCLC cell sensitivity to MP‐470.

Finally, we evaluated the clonogenic potential of H460 cells in the presence of MP‐470. We found that MP‐470 caused a significant reduction in the surviving fraction of H460 βIII_SH4_ cells compared to H460 Ctrl_SH2_ cells. Furthermore, this phenotype was reversed in the βIII‐tubulin rescue cells (Figure 2C), demonstrating that βIII‐tubulin also plays a role in the clonal expansion of H460 cells in response to MP‐470 treatment.

### 3.3 MP‐470 inhibits NSCLC cell proliferation following βIII‐tubulin suppression

**FIGURE 3:**
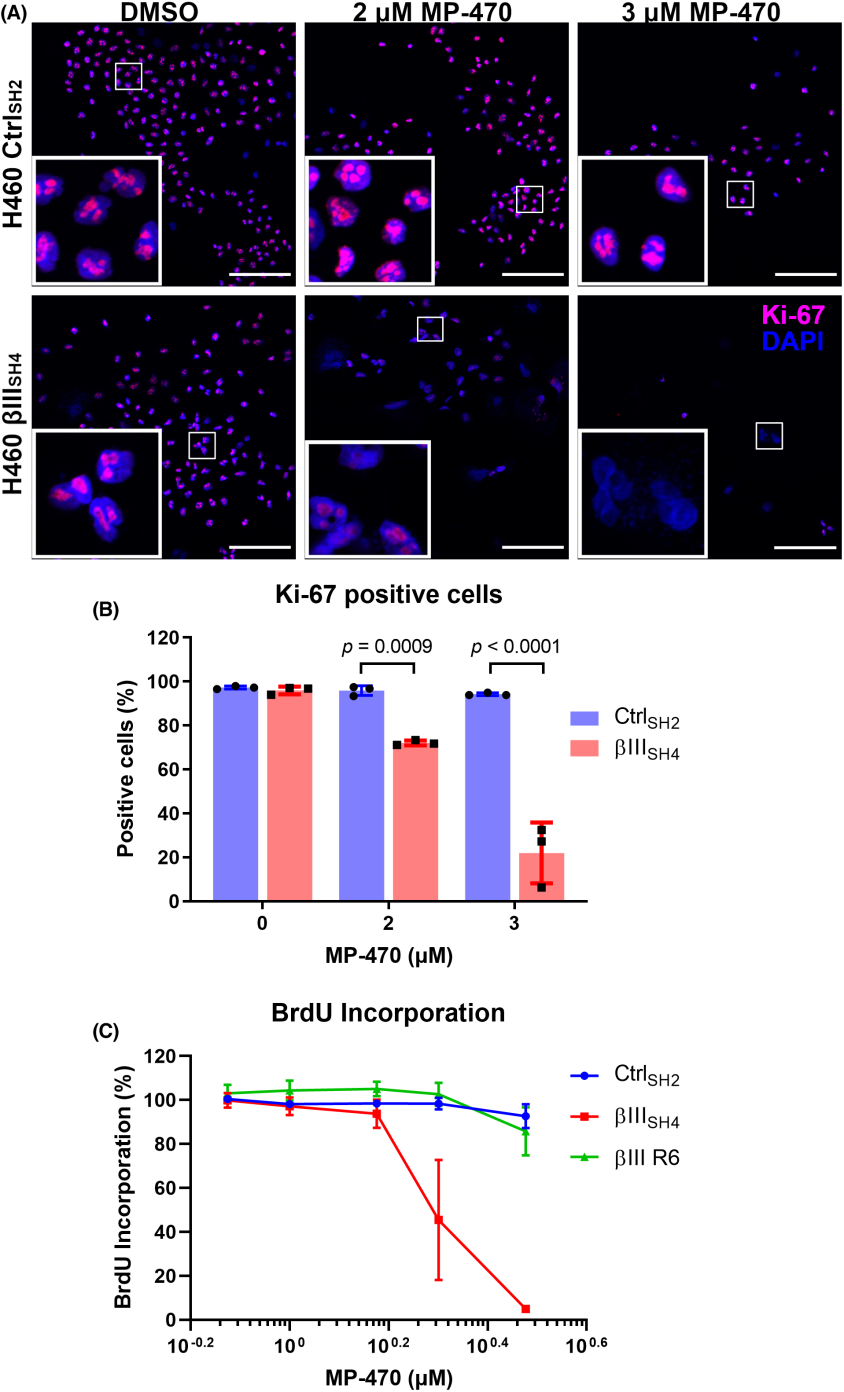
MP‐470 inhibits H460 cell proliferation in 2D cell cultures following βIII‐tubulin suppression. (A) Representative immunofluorescence images of βIII‐tubulin knockdown (βIII_SH4_) and Control (Ctrl_SH2_) H460 cells treated with MP‐470 or DMSO and stained for the proliferation marker Ki‐67 (red) and nuclei (blue). Images are 20× magnification, insets are 5x magnifications of the regions shown. Scale bars = 100 μm. (B) Image quantification showing percentage of Ki‐67‐positive H460 cells following treatment with MP‐470 or DMSO (*n* = 3, mean ± SD, each point represents one biological replicate; Shapiro–Wilk test to confirm normality followed by a 2‐way ANOVA with Sidak's multiple comparison test, interaction Ctrl_SH2_ vs. βIII_SH4_ F (2, 12) = 58.32, *p* < 0.0001). (C) BrdU‐Incorporation ELISA showing reduced cell proliferation in MP‐470 treated βIII‐tubulin knockdown (βIII_SH4_) compared to control (Ctrl_SH2_) H460 cells. Phenotype is then restored by re‐expression of βIII‐tubulin (*n* = 3, mean ± SD; MP‐470 concentration was log‐transformed; comparison via nonlinear regression analysis, Ctrl_SH2_ vs. βIII_SH4_ F (2, 36) = 145.7, *p* < 0.0001, Ctrl_SH2_ vs. βIII R6 F (2, 36) = 2.195, *p* = 0.1260, βIII_SH4_ vs. βIII R6 F (2, 36) = 109.5, *p* < 0.0001).

Clonal expansion is a result of the replicative ability of cells and hence, cell proliferation. To investigate whether MP‐470 may be exerting an anti‐proliferative effect, MP‐470 treated H460 Ctrl_SH2_ and H460 βIII_SH4_ cells were immunostained for expression of Ki‐67, a well‐established marker of proliferating cells. We found that, while Ki‐67 was visible in the nuclei of DMSO treated H460 βIII_SH4_ cells, staining was significantly reduced in H460 βIII_SH4_ cells treated with MP‐470 (Figure 3A,B), suggesting cell proliferation was inhibited by MP‐470 in these cells. In contrast, Ki‐67 was not inhibited in H460 Ctrl_SH2_ cells treated with MP‐470 or with DMSO alone.

We also measured incorporation of BrdU into newly synthesised DNA, another commonly used method to detect replicating cells. We found that BrdU incorporation was reduced in βIII‐tubulin knockdown H460 cells following treatment with MP‐470 compared to control cells (Figure 3C; Figure S2A). In addition, we also found that re‐expression of βIII‐tubulin protein restored BrdU incorporation following MP‐470 treatment (Figure 3C), suggesting βIII‐tubulin plays a role in mediating the anti‐proliferative effect of MP‐470 in H460 cells.

### 3.4 MP‐470 inhibits H460 cell proliferation in 3D tumour cell spheroids following βIII‐tubulin suppression

**FIGURE 4:**
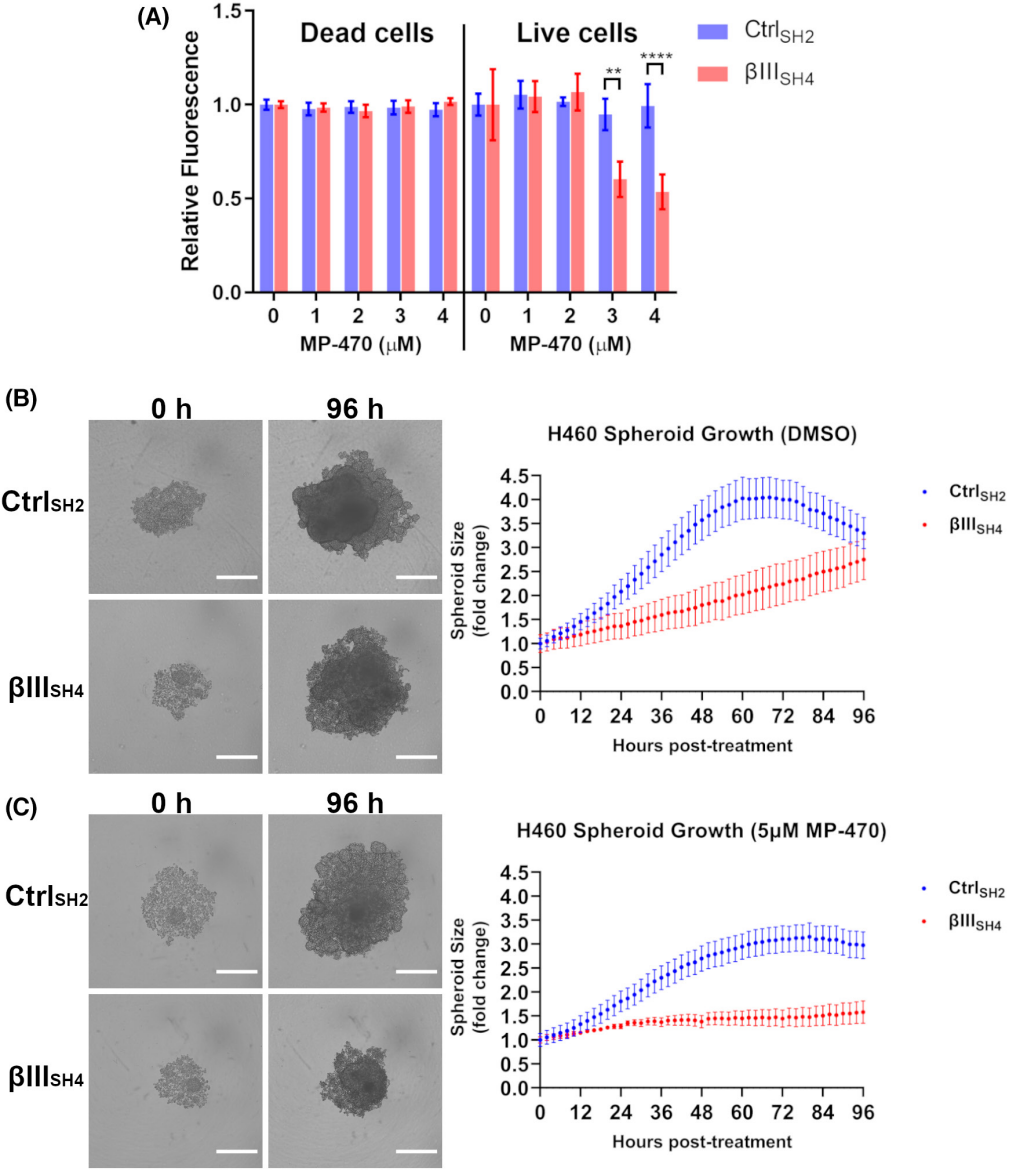
MP‐470 inhibits H460 cell proliferation in 3D tumour cell spheroids following βIII‐tubulin suppression. (A) MP‐470‐treated Control (Ctrl_SH2_) and βIII‐tubulin knockdown (βIII_SH4_) H460 cells were co‐labelled to quantify relative numbers of dead cells (left) and live cells (right) (*n* = 3, mean ± SD; SD generated with error propagation; Shapiro–Wilk test to confirm normality followed by a 2‐way ANOVA with Sidak's multiple comparison test, interaction Ctrl_SH2_ vs. βIII_SH4_ dead cells F (4, 20) = 0.9415, *p* = 0.4604, interaction Ctrl_SH2_ vs. βIII_SH4_ live cells F (4, 20) = 8.131, *p* = 0.0005; **p = 0.0020, ****p < 0.0001). (B) Representative images and growth curves of Control (Ctrl_SH2_) and βIII‐tubulin knockdown (βIII_SH4_) H460 cells grown as 3D tumour spheroids treated with DMSO. Images are 4x magnification. Scale bars = 500 μm. (*n* = 8 spheroids analysed per condition, mean ± SEM, D'Agostino & Pearson test to confirm normality followed by a 2‐way ANOVA with Sidak's multiple comparison test, interaction F(48, 686) = 1.729, *p* = 0.0021); multiple comparison observed significance between 44 and 76 h where *p* values ranged between 0.0452 and 0.0026, no significant difference was seen from 78 to 96 h. (C) Representative images and real‐time growth analysis of Control (Ctrl_SH2_) and βIII‐tubulin knockdown (βIII_SH4_) H460 cells grown as 3D tumour spheroids treated with 5 μM MP‐470. Images are 4× magnification. Scale bars = 500 μm. (*n* = 8 spheroids analysed per condition, mean ± SEM, D'Agostino & Pearson test to confirm normality followed by a 2‐way ANOVA with Sidak's multiple comparison test, interaction F(48, 686) = 4.279, *p* < 0.0001; multiple comparison observed significance from 38 h where *p* values ranged from 0.0351 to less than 0.0001).

To evaluate whether MP‐470 may be causing cell death in addition to inhibiting proliferation, we performed a multiplex assay whereby cells are co‐labelled to differentiate between live and dead cells. We found no change in the number of dead cells in either H460 Ctrl_SH2_ or H460 βIII_SH4_ cells following MP‐470 treatment (Figure 4A; left), suggesting cell death was not occurring in these cells. In contrast, significantly fewer live cells were detected among H460 βIII_SH4_ cells treated with increasing concentrations of MP‐470 compared to H460 Ctrl_SH2_ cells (Figure 4A; right), supporting our earlier finding of reduced cell proliferation. To further rule out cell death, we examined apoptosis. To do this, we measured activity of Caspase‐9, a well‐characterised initiator caspase and apoptosis marker. We found that Caspase‐9 activity was not elevated in either H460 Ctrl_SH2_ or H460 βIII_SH4_ cells following MP‐470 treatment at any of the concentrations we tested (Figure S2B). To determine if MP‐470 was causing senescence in these cells, we stained H460 βIII_SH4_ cells for β‐galactosidase activity, a biomarker for cellular senescence. While we were able to detect β‐galactosidase activity using a senescence positive control (H_2_O_2_ treatment), we were unable to detect senescence in H460 βIII_SH4_ cells following MP‐470 treatment (Figure S2C), suggesting that MP‐470 does not induce senescence in these cells. Together, these data show that MP‐470 selectively inhibits cell proliferation in H460 cells following βIII‐tubulin suppression, rather than inducing cell death or senescence. Finally, cell cycle progression as analysed by flow cytometry, identified that MP‐470 was inducing a significant G0/G_1_ delay (*p* = 0.0451) in H460 βIII_SH4_ cells compared to the H460 βIII_SH4_ non treated cells (Figure S2D). This was accompanied by significant decreases in S phase (*p* = 0.0261) and G_2_M phase (*p* = 0.0451) in the MP‐470 treated H460 βIII_SH4_ cells compared to the H460 βIII_SH4_ non treated cells. No significant impact was observed on the cycling of H460 Ctrl_SH2_ cells (Figure S2D). This is consistent with another study that showed that MP‐470‐mediated G0/G_1_ arrest may be cancer‐type specific.^29^ Future studies are required to clearly elucidate the impact and relationship between MP‐470, βIII‐tubulin and cell cycle.

To examine the anti‐proliferative effects of MP‐470 in a more physiologically relevant model of NSCLC, we measured the effect of MP‐470 on the growth of 3D tumour spheroids. To do this, H460 Ctrl_SH2_ and βIII_SH4_ cells were allowed to form 3D spheroids before being treated with MP‐470 and imaged over time. Real‐time monitoring of spheroid growth revealed that H460 βIII_SH4_ spheroids grew at a slower rate than H460 Ctrl_SH2_ spheroids (Figure 4B), an observation consistent with our previous work.^15^ We also found that this effect was significantly enhanced by the addition of MP‐470, such that MP‐470 treated βIII_SH4_ cells developed significantly smaller spheroids over the course of the assay compared to Ctrl_SH2_ spheroids (Figure 4C). This result further demonstrates that MP‐470 combined with βIII‐tubulin suppression has an anti‐proliferative effect on H460 cells grown as 3D tumour spheroids in vitro.

### 3.5 MP‐470 combined with βIII‐tubulin suppression acts via the ERK signalling pathway to inhibit cell proliferation in c‐Met expressing H460 cells

**FIGURE 5:**
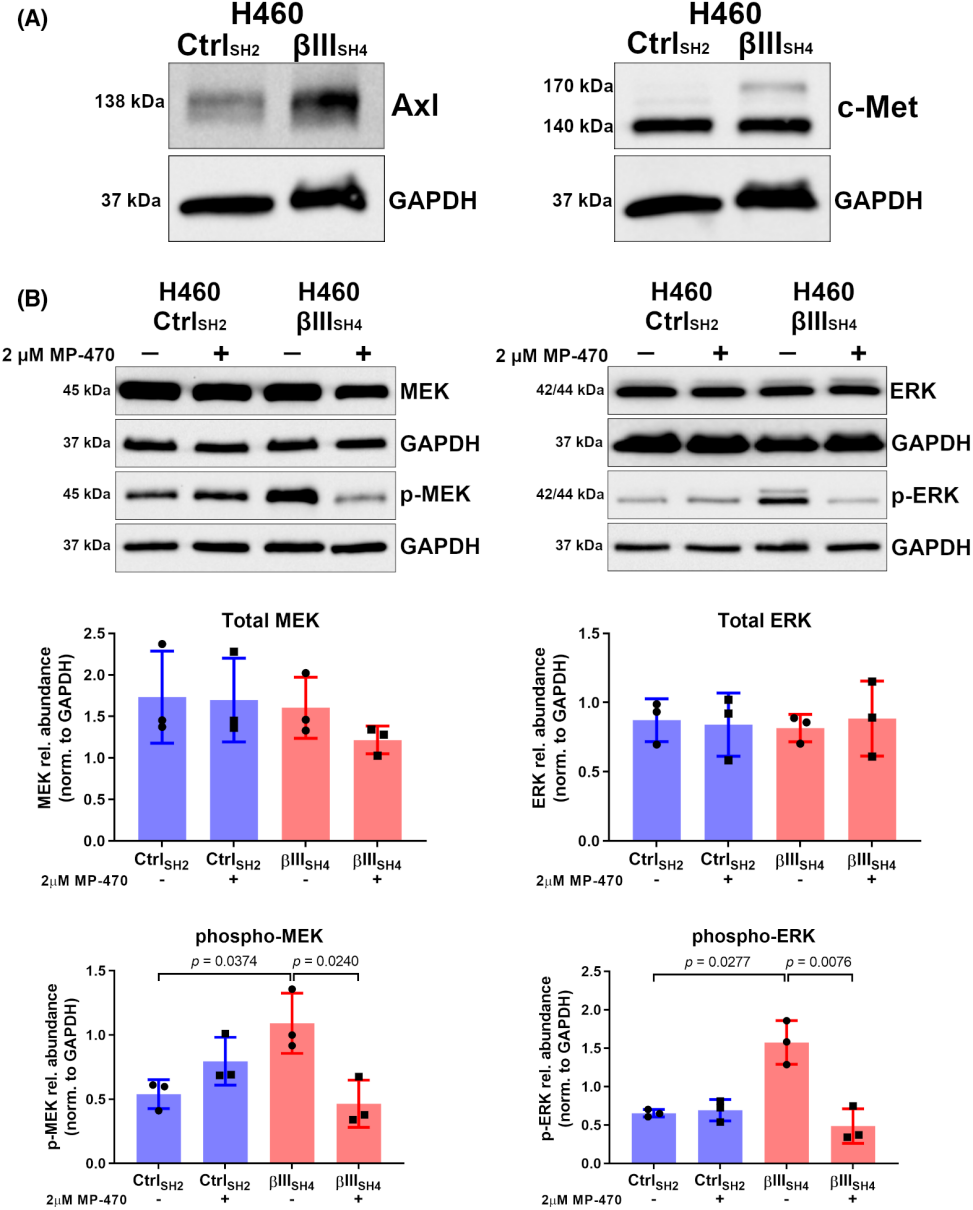
MP‐470 combined with βIII‐tubulin suppression reduces ERK signalling. (A) Representative western blots confirming protein expression of Axl (left) and c‐Met (right) in Control (Ctrl_SH2_) and βIII‐tubulin knockdown (βIII_SH4_) H460 cells. (B) Representative western blots and quantitation by densitometry showing total MEK, total ERK, phospho‐MEK, and phospho‐ERK levels in Control (Ctrl_SH2_) and βIII‐tubulin knockdown (βIII_SH4_) H460 cells following MP‐470 treatment (*n* = 3, mean ± SD, each point represents one biological replicate; Shapiro–Wilk test to confirm normality followed by Student's t‐test using either Welch's correction or Mann–Whitney's test).

To better understand the mechanism by which MP‐470 inhibits cell proliferation, we investigated the expression of its targets and the downstream signalling pathways that contribute to this effect. It has been reported that MP‐470 is a multi‐targeted inhibitor of the receptor tyrosine kinases Flt3, c‐Kit, Axl, c‐Met, PDGFRα/β, and c‐Ret.^29^, ^30^, ^31^ To identify which of these purported targets are expressed in our NSCLC cell‐lines, we first investigated their expression via RT‐PCR. We found that H460 cells expressed *AXL, MET*, and *PDGFRβ* mRNA, as did A549 cells with the inclusion of *PDGFRα* (Figure S3A). We then confirmed these results at the protein level via western blotting. While we found that expression of both Axl and c‐Met was detected in H460 cells (Figure 5A) and A549 cells (Figure S3B), we did not detect expression of PDGFRα or PDGFRβ proteins in either H460 (Figure S3C) or A549 cells (Figure S3D). Interestingly, aberrant expression of both Axl and c‐Met has been associated with more aggressive forms of NSCLC,^32^, ^33^ where high expression of either has been associated with poor patient prognosis. Additionally, both Axl and c‐Met share some degree of cross talk with one another.^34^ Having validated these MP‐470 targets as being expressed by our cells, it suggested to us that the receptor tyrosine kinases Axl or c‐Met were potential candidates involved in our cell models' responses to MP‐470. This, combined with their previously established roles in NSCLC, meant these became the focus of the next part of our study in the context of βIII‐tubulin expression in NSCLC.

Next, we explored the signalling pathways downstream of the Axl or c‐Met receptors, and how these are impacted by MP‐470. Initially, we examined the AKT pathway, as this pathway has known roles in both cell proliferation and survival.^35^ Furthermore, we have previously demonstrated that βIII‐tubulin contributes to activation of the AKT pathway in H460 cells.^15^ We therefore postulated that βIII‐tubulin might also influence H460 cell response to MP‐470 via this pathway. The AKT pathway is tightly controlled by the tumour‐suppressor PTEN, which acts upstream of AKT as a negative regulator.^36^ We hypothesised that if H460 βIII_SH4_ cell sensitivity to MP‐470 was mediated by diminished AKT activation, then removing PTEN's inhibition of this pathway would reverse this phenotype. To test this hypothesis, we used siRNA to knockdown expression of PTEN in H460 βIII_SH4_ cells (Figure S4A), and measured cell viability in response MP‐470 treatment. Interestingly, we did not see a reversal of the decrease in cell viability in PTEN knockdown cells compared to control (Figure S4B), suggesting an alternative signalling pathway was involved in H460 βIII_SH4_ cell sensitivity to MP‐470.

Another signalling pathway that has been shown to play a role in cell proliferation downstream of receptor tyrosine kinase activation is the MEK/ERK pathway.^37^, ^38^ Phosphorylation of ERK downstream of MEK has been shown to stimulate proliferation in a variety of different cell‐types including NSCLC.^39^, ^40^ We hypothesised that perturbed MEK/ERK signalling might explain the observed reduction in cell proliferation following MP‐470 treatment. To investigate this, we treated H460 Ctrl_SH2_ and H460 βIII_SH4_ cells with MP‐470 and probed cell lysates for phosphorylated MEK and ERK (p‐MEK and p‐ERK, respectively). We found that H460 βIII_SH4_ cells expressed higher levels of p‐MEK and p‐ERK compared to Ctrl_SH2_ cells, and upon treating these cells with MP‐470 we found that, while total MEK–ERK levels were unaffected, there was a significant reduction in p‐MEK and p‐ERK compared to DMSO‐treated cells (Figure 5B), suggesting a key role for the MEK–ERK pathway in mediating H460 βIII_SH4_ cell response to MP‐470.

**FIGURE 6:**
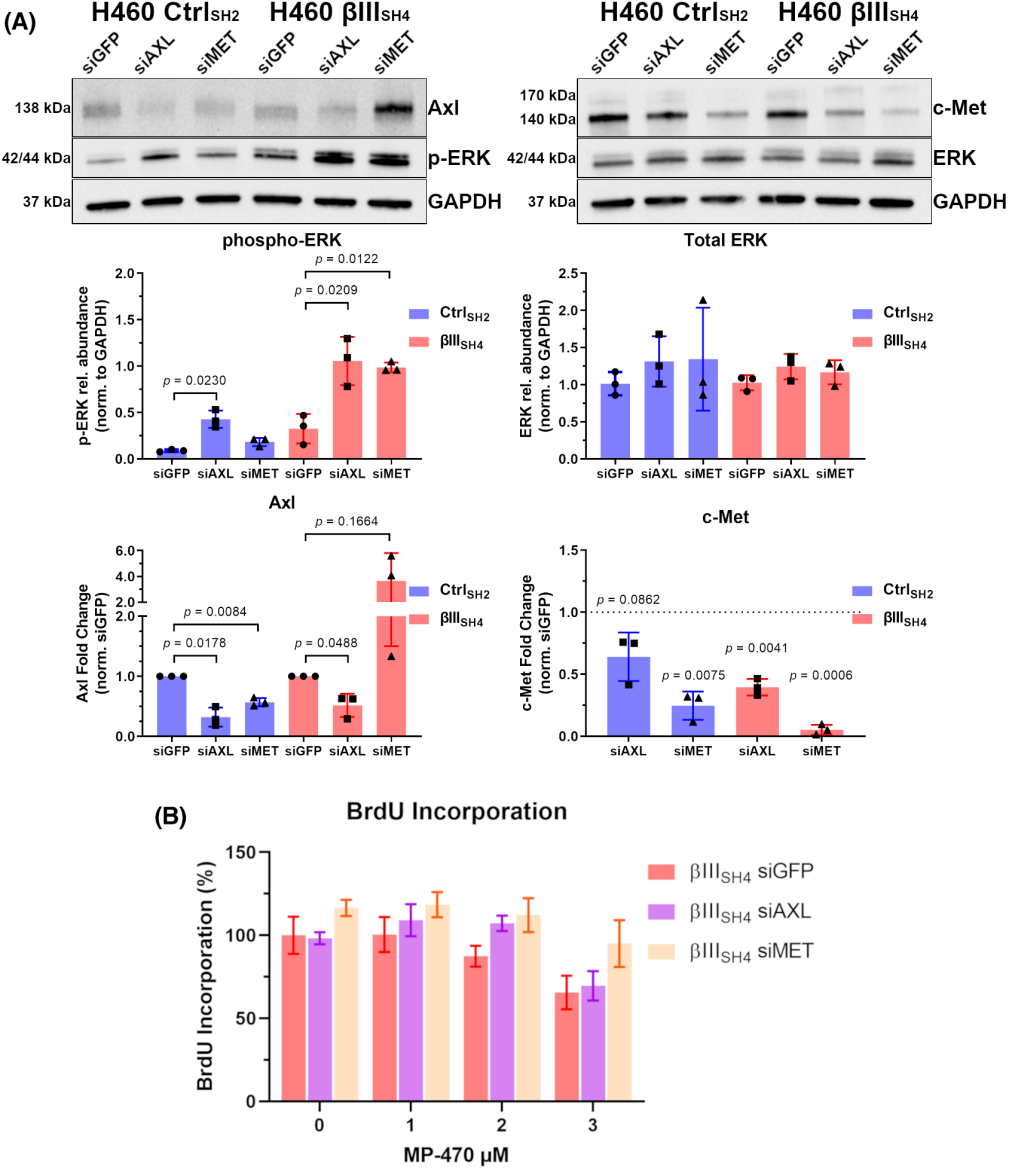
MP‐470 combined with βIII‐tubulin suppression inhibits cell proliferation in c‐Met expressing H460 cells. (A) Representative western blot and quantitation by densitometry showing phospho‐ERK, total ERK, Axl and c‐Met levels following Axl or c‐Met siRNA knockdown in Control (Ctrl_SH2_) and βIII‐tubulin knockdown (βIII_SH4_) H460 cells. Phospho‐ERK and total ERK presented as relative abundance (*n* = 3, mean ± SD, each point represents one biological replicate; Shapiro–Wilk test to confirm normality followed by Student's t‐test using either Welch's correction or Mann–Whitney's test). Axl and c‐Met levels presented as fold change Axl/c‐Met expression in siGFP transfected cells where relative abundance (normalised to GAPDH) was normalised to cells of the same background transfected with siGFP (*n* = 3, mean ± SD, each point represents one biological replicate; dotted line represents siGFP transfected cells; significant assessed using One Sample t test compared to a theoretical mean of 1). (B) BrdU‐Incorporation ELISA showing that the reduced cell proliferation of MP‐470‐treated βIII_SH4_ cells is partially restored by siRNA knockdown of c‐Met, but not Axl. (*n* = 3, mean ± SD; SD generated with error propagation; comparison via nonlinear regression analysis, siGFP vs. siAXL F (2, 20) = 2.814, *p* = 0.0838, siGFP vs. siMET F (2, 20) = 4.649, *p* = 0.0220, siAXL vs. siMET F (2, 20) = 2.530, *p* = 0.1048).

To further investigate ERK signalling in the context of MP‐470 target expression in our cell models, we used siRNA to knock down expression of Axl or c‐Met and then measured the levels of p‐ERK. In both H460 Ctrl_SH2_ and H460 βIII_SH4_ cells. Knockdown of AXL resulted in an elevation of p‐ERK expression compared to cells transfected with control siRNA (siGFP) (Figure 6A). p‐ERK expression was also increased in H460 βIII_SH4_ cells transfected with siMET (Figure 6A). In contrast, H460 Ctrl_SH2_ cells with siRNA knockdown of MET displayed no significant alteration to p‐ERK levels, and no changes in total ERK levels across any of the groups (Figure 6A). Interestingly, unexpected changes to the levels Axl and c‐Met were also observed, as c‐Met expression was reduced in H460 βIII_SH4_ cells with AXL siRNA knockdown (decreasing trend in H460 Ctrl_SH2_ cells) and H460 Ctrl_SH2_ cells with MET siRNA knockdown displayed reduced Axl expression (Figure 6A). In contrast to the H460 Ctrl_SH2_ cells, H460 βIII_SH4_ cells with MET siRNA knockdown displayed an increasing trend in Axl expression (Figure 6A). These data suggest that Axl is involved with the regulation of ERK activation in H460 cells, and that reduced βIII‐tubulin expression combined with reduced c‐Met expression is inducing ERK phosphorylation.

Finally, to determine if MP‐470 was inhibiting cell proliferation through Axl or c‐Met, we measured BrdU incorporation in H460 βIII_SH4_ cells co‐treated with MP‐470 and Axl or c‐Met siRNA. Interestingly, we found that knockdown of c‐Met, but not Axl partially restored the proliferative capacity of H460 βIII_SH4_ cells following MP‐470 treatment compared to siGFP knockdown cells (Figure 6B), suggesting that the expression of c‐Met, but not Axl, is playing an important role in mediating the anti‐proliferative effect of MP‐470 activity in our cell models of NSCLC (See Figure S5 for detailed schematic of proposed model).

## 4 DISCUSSION

Clinical studies have identified high levels of βIII‐tubulin as a poor prognostic indicator in NSCLC.^8^, ^41^ Numerous studies from our lab and those of others have identified a role for βIII‐tubulin in drug resistance in a range of epithelial cancers including NSCLC.^3^, ^7^, ^8^, ^9^, ^10^, ^11^ Our research has also revealed a novel role for this tubulin isotype in regulating the PTEN/AKT signalling axis.^15^ Here, we performed a high‐throughput kinase screen against NSCLC cells with suppressed βIII‐tubulin to identify kinases that could sensitise these cells to this combinatorial approach. We identified for the first time, that Amuvatinib (MP‐470) activity is enhanced by suppression of βIII‐tubulin in NSCLC cells.

Receptor tyrosine kinases (RTKs) have been shown to play a critical role in the development and progression of many cancers, and several RTKs including c‐Met have been identified as therapeutic targets in NSCLC.^42^ RTKs generally undergo autophosphorylation, which in turn promotes the recruitment of downstream effector proteins leading to the activation of multiple signal cascades. Among these, the three‐tiered MAPK cascade consisting of RAF, MEK, and ERK is frequently dysregulated in many malignancies including NSCLC.^43^ It has been shown that ERK activation can mediate NSCLC cell death in response to c‐Met inhibitors,^44^ and MEK inhibitors have been shown to be effective against *MET*‐amplified NSCLC cells.^45^ Here, we show that Amuvatinib selectively inhibits cell proliferation in βIII‐tubulin supressed cells by disrupting MAPK signalling downstream of c‐Met, highlighting a novel approach for targeting this important signalling axis in NSCLC. Interestingly, when investigating MAPK signalling in our cell models of NSCLC, we found that H460 βIII‐tubulin suppressed cells expressed higher basal levels of both p‐MEK and p‐ERK, suggesting a possible, as‐yet unexplored, role for βIII‐tubulin in impacting MAPK signalling. Additionally, a recent study found that βIII‐tubulin suppression decreased MYC levels, which in turn sensitised pancreatic cancer cells to ERK inhibition.^46^ Whether this pathway is active in our NSCLC cells remains to be investigated.

Through knocking down c‐Met expression in βIII‐tubulin‐suppressed cells, our study identified a potential compensatory mechanism to maintain some aspect of the proliferative capacity of the cells through increased Axl expression. This trending increase in Axl expression may also account for the Amuvatinib resistance gained in the βIII‐tubulin and c‐Met suppressed cells, as c‐Met is more susceptible to Amuvatinib inhibition than Axl.^30^ This potential compensatory mechanism between c‐MET and Axl does not appear to extend to the cells with unaltered βIII‐tubulin levels, as knockdown of c‐Met appears to lead to a reduction in Axl expression. This is consistent with data reported in human bladder cancer cells, supporting the notion that c‐Met may have some transcriptional control over Axl expression^47^; how the loss of βIII‐tubulin potentially reverses this effect, and whether βIII‐tubulin plays a role in regulating phosphorylation of c‐MET requires further investigation. Targeting of Axl expression and subsequent reduction in c‐Met expression suggests cross‐talk between these pathways in βIII‐tubulin suppressed cells. The finding that βIII‐tubulin and Axl suppressed cells were still susceptible to Amuvatinib, despite the reduction in c‐Met expression, may be due the low levels of c‐Met present. It is not clear why targeting of Axl led to this impact on c‐Met expression, and the likely impact of targeting c‐Met on the expression of Axl, warrants further investigation.

The differential expression of tubulin isotypes in cancers, such as overexpression of βI and βIII‐tubulin in breast and lung cancers, and highly expressed βVI‐tubulin in normal blood cells has triggered great interest in designing specific tubulin isotype‐binding anticancer therapies.^48^ Small‐molecule targeting of specific tubulins however has been met with challenges, mostly due to similarities in protein structure and overlapping drug‐binding domains between isotypes. While some progress has been made with analogues of Taxol,^49^ and novel colchicine derivatives,^50^ another approach is to use siRNA to therapeutically silence the expression of individual tubulin genes. Despite the potential of siRNA therapies, there are hurdles limiting their clinical application such as poor transport across biological barriers, limited cellular uptake, degradation, and rapid clearance.^51^ Nanotechnology has come to the forefront with recent developments of highly effective delivery vehicles for siRNA‐based therapies^52^ and significant progress has been made in this area with RNAi‐based nanomedicines having undergone or currently in clinical trials for a variety of different cancers.^53^, ^54^ Importantly, we have previously successfully silenced βIII‐tubulin using nanoparticles to deliver siRNA to pancreatic tumours^55^ and more recently, βIII‐tubulin siRNA alone or in combination with a chemotherapy drug in lung cancer cells in vitro and *in vivo*.^56^, ^57^ This, in conjunction with our findings here using multiple NSCLC cell lines in 2D and 3D spheroid cultures, shows that in principle, silencing of βIII‐tubulin using siRNA‐nanoparticles combined with small‐molecule kinase inhibitors could be developed as a therapeutic strategy for NSCLC. One of the limitations of this study is its reliance on in vitro cell culture models of NSCLC, and future studies using clinically relevant mouse models of lung cancer are needed to validate the therapeutic potential of combing gene silencing βIII‐tubulin nanodrugs with Amuvatinib for the treatment of non‐small cell lung cancer. Finally, our findings here could highlight a potential personalised treatment for a subset of patients who, upon biopsy are found to have tumours expressing high c‐Met but low βIII‐tubulin and may therefore benefit from Amuvatinib.

## AUTHORS CONTRIBUTIONS

Conceptualisation: SB, WT, TD, GMA, JAM, MK; Methodology: SB, WT, EG‐A, ZM, TF; Formal analysis: SB, AD; Investigation: SB, AD, WT, TD, LM; Writing – original draft: SB; Writing ‐ review & editing: SB, JAM, MK; Project administration: MK; Funding acquisition: JAM, MK.

## FUNDING INFORMATION

This work was supported by the Children's Cancer Institute, which is affiliated with the University of New South Wales (UNSW Sydney), and the Sydney Children's Hospital Network, and by grants from the National Health and Medical Research Council (Program Grant APP1091261; Principal Research Fellowship APP1119152 to MK; and Project Grant APP1144113 to JM) and Cancer Australia (1,141,485 to MK and JM).

## CONFLICT OF INTEREST

The authors declare no competing interests.

## ETHICAL APPROVAL STATEMENT

Not Applicable.

## Supporting information


Appendix S1

## Data Availability

Data available on request from the authors.
