# Supplementary material for: βIII‐tubulin suppression enhances the activity of Amuvatinib to inhibit cell proliferation in c‐Met positive non‐small cell lung cancer cells
Source: Cancer Med. 2022 Aug 10;12(4):4455–71. doi: 10.1002/cam4.5128 (PMC9972117; doi:10.1002/cam4.5128)
Supplement: Supplementary file 1 — Appendix S1 [file CAM4-12-4455-s001.pptx]

## Slide 1
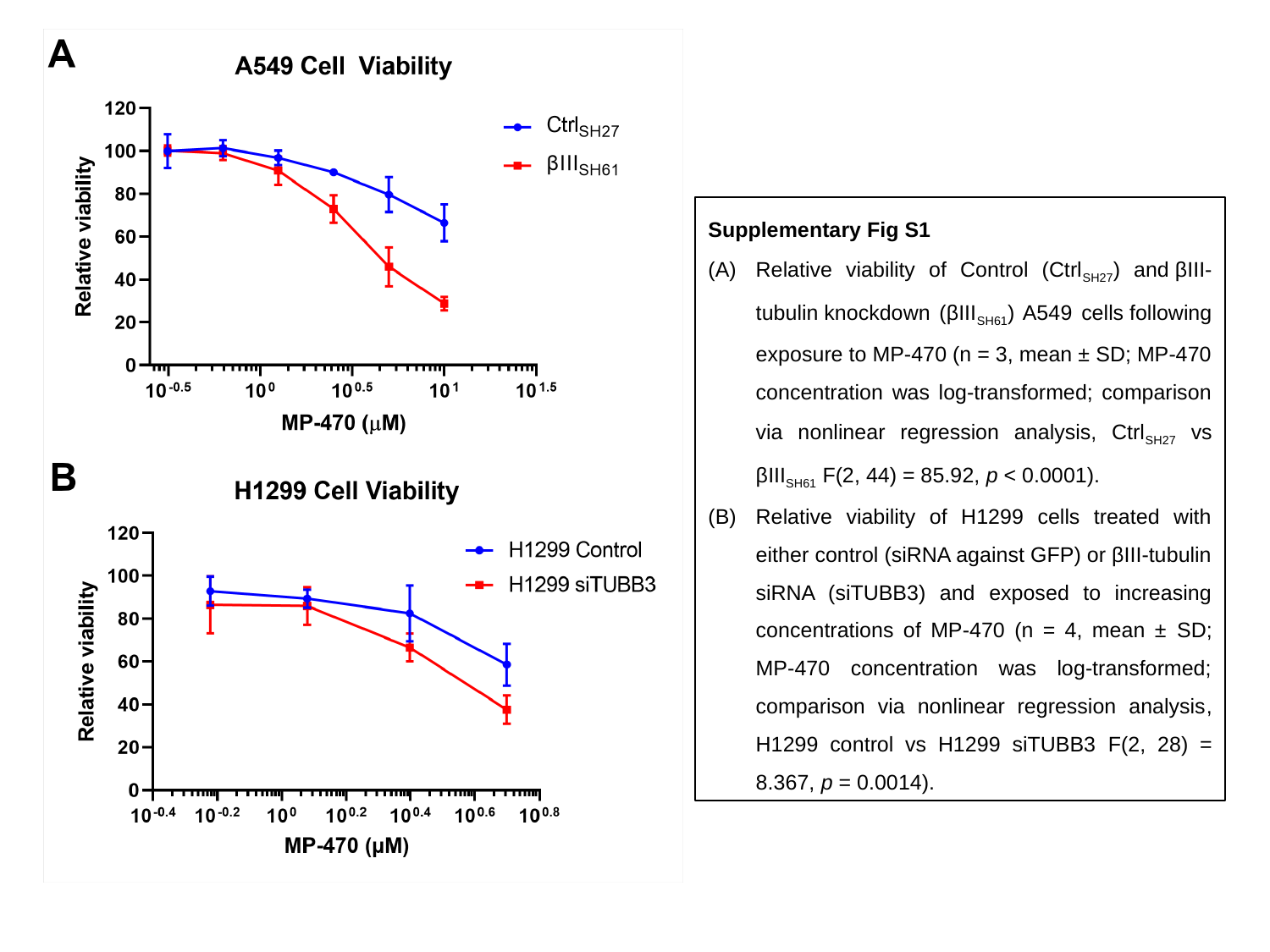

Supplementary Fig S1
Relative viability of Control (CtrlSH27) and βIII-tubulin knockdown (βIIISH61) A549 cells following exposure to MP-470 (n = 3, mean ± SD; MP-470 concentration was log-transformed; comparison via nonlinear regression analysis, CtrlSH27 vs βIIISH61 F(2, 44) = 85.92, p < 0.0001).
Relative viability of H1299 cells treated with either control (siRNA against GFP) or βIII-tubulin siRNA (siTUBB3) and exposed to increasing concentrations of MP-470 (n = 4, mean ± SD; MP-470 concentration was log-transformed; comparison via nonlinear regression analysis, H1299 control vs H1299 siTUBB3 F(2, 28) = 8.367, p = 0.0014).

## Slide 2
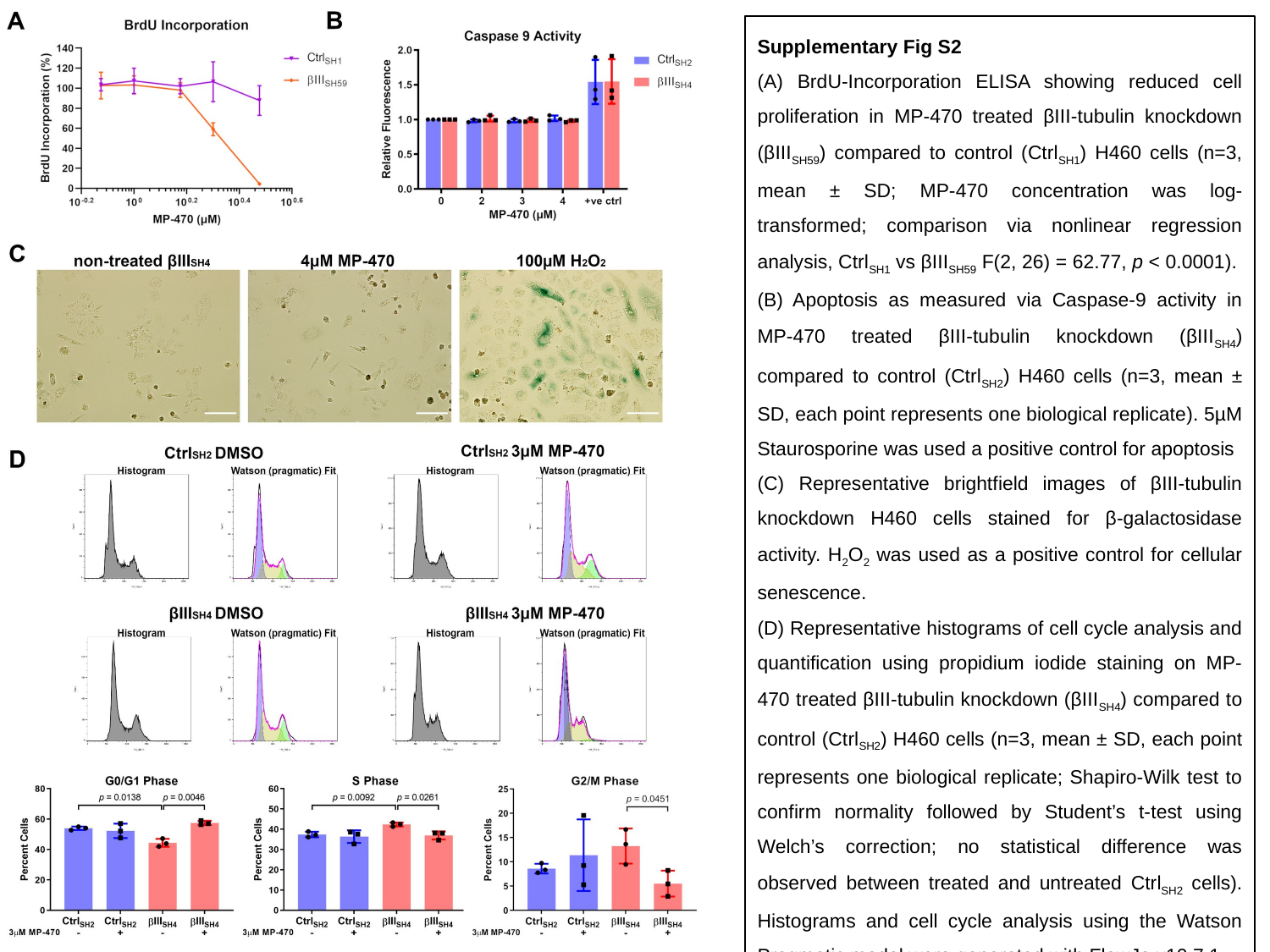

Supplementary Fig S2
(A) BrdU-Incorporation ELISA showing reduced cell proliferation in MP-470 treated βIII-tubulin knockdown (βIIISH59) compared to control (CtrlSH1) H460 cells (n=3, mean ± SD; MP-470 concentration was log-transformed; comparison via nonlinear regression analysis, CtrlSH1 vs βIIISH59 F(2, 26) = 62.77, p < 0.0001).
(B) Apoptosis as measured via Caspase-9 activity in MP-470 treated βIII-tubulin knockdown (βIIISH4) compared to control (CtrlSH2) H460 cells (n=3, mean ± SD, each point represents one biological replicate). 5µM Staurosporine was used a positive control for apoptosis
(C) Representative brightfield images of βIII-tubulin knockdown H460 cells stained for β-galactosidase activity. H2O2 was used as a positive control for cellular senescence.
(D) Representative histograms of cell cycle analysis and quantification using propidium iodide staining on MP-470 treated βIII-tubulin knockdown (βIIISH4) compared to control (CtrlSH2) H460 cells (n=3, mean ± SD, each point represents one biological replicate; Shapiro-Wilk test to confirm normality followed by Student’s t-test using Welch’s correction; no statistical difference was observed between treated and untreated CtrlSH2 cells). Histograms and cell cycle analysis using the Watson Pragmatic model were generated with FlowJo v10.7.1.

## Slide 3
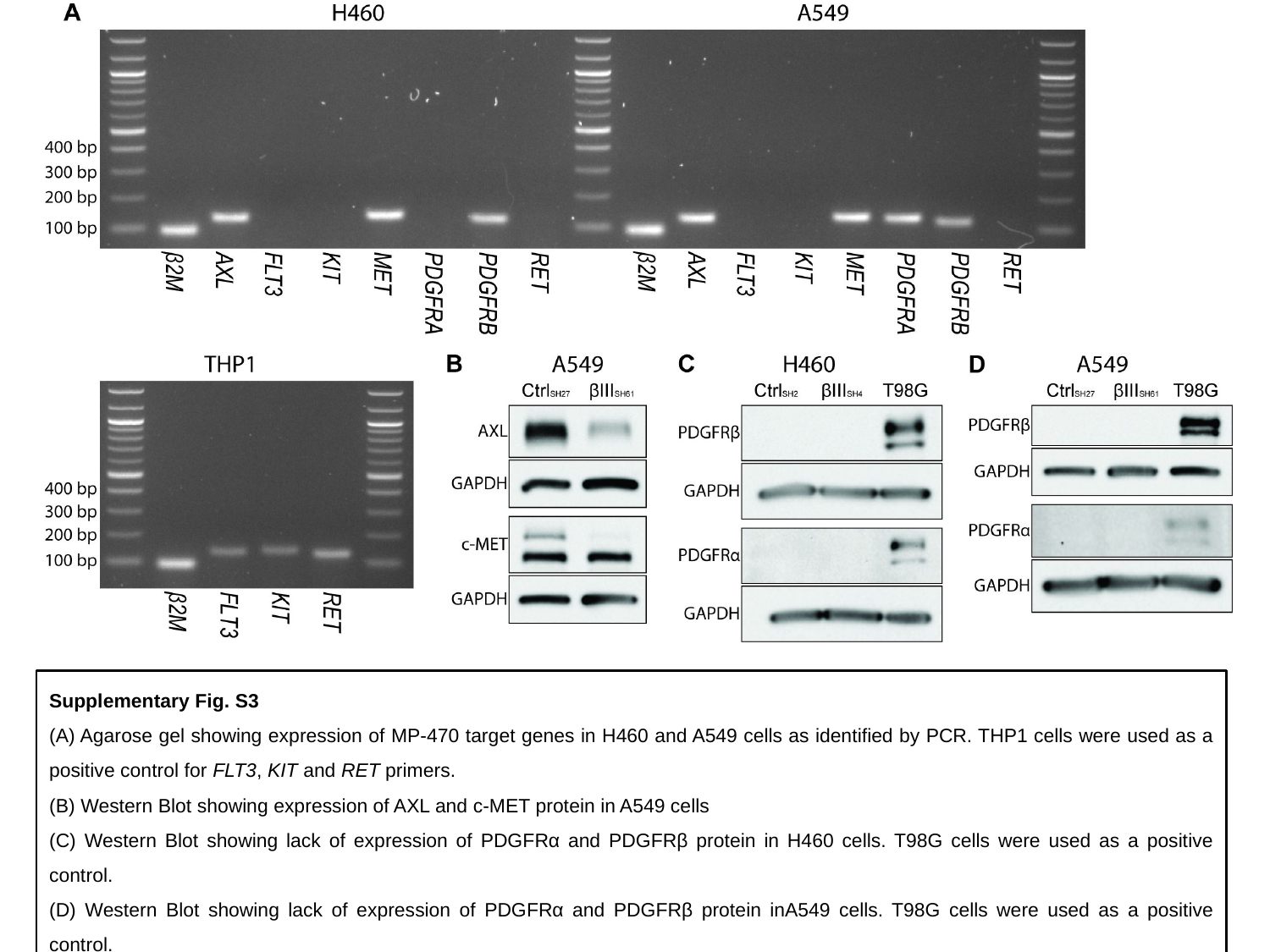

Supplementary Fig. S3
(A) Agarose gel showing expression of MP-470 target genes in H460 and A549 cells as identified by PCR. THP1 cells were used as a positive control for FLT3, KIT and RET primers.
(B) Western Blot showing expression of AXL and c-MET protein in A549 cells
(C) Western Blot showing lack of expression of PDGFRα and PDGFRβ protein in H460 cells. T98G cells were used as a positive control.
(D) Western Blot showing lack of expression of PDGFRα and PDGFRβ protein inA549 cells. T98G cells were used as a positive control.

## Slide 4
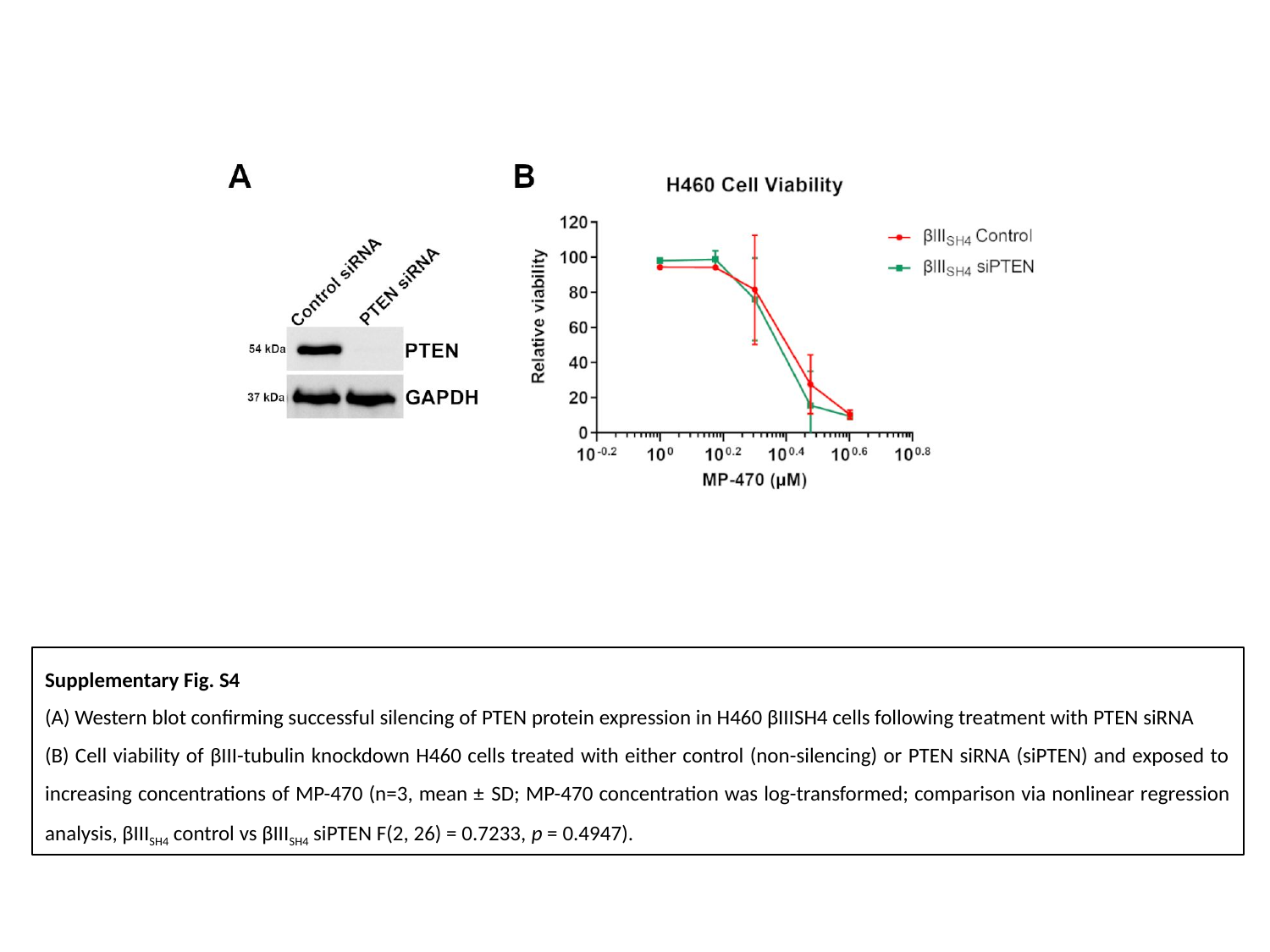

Supplementary Fig. S4
(A) Western blot confirming successful silencing of PTEN protein expression in H460 βIIISH4 cells following treatment with PTEN siRNA
(B) Cell viability of βIII-tubulin knockdown H460 cells treated with either control (non-silencing) or PTEN siRNA (siPTEN) and exposed to increasing concentrations of MP-470 (n=3, mean ± SD; MP-470 concentration was log-transformed; comparison via nonlinear regression analysis, βIIISH4 control vs βIIISH4 siPTEN F(2, 26) = 0.7233, p = 0.4947).

## Slide 5
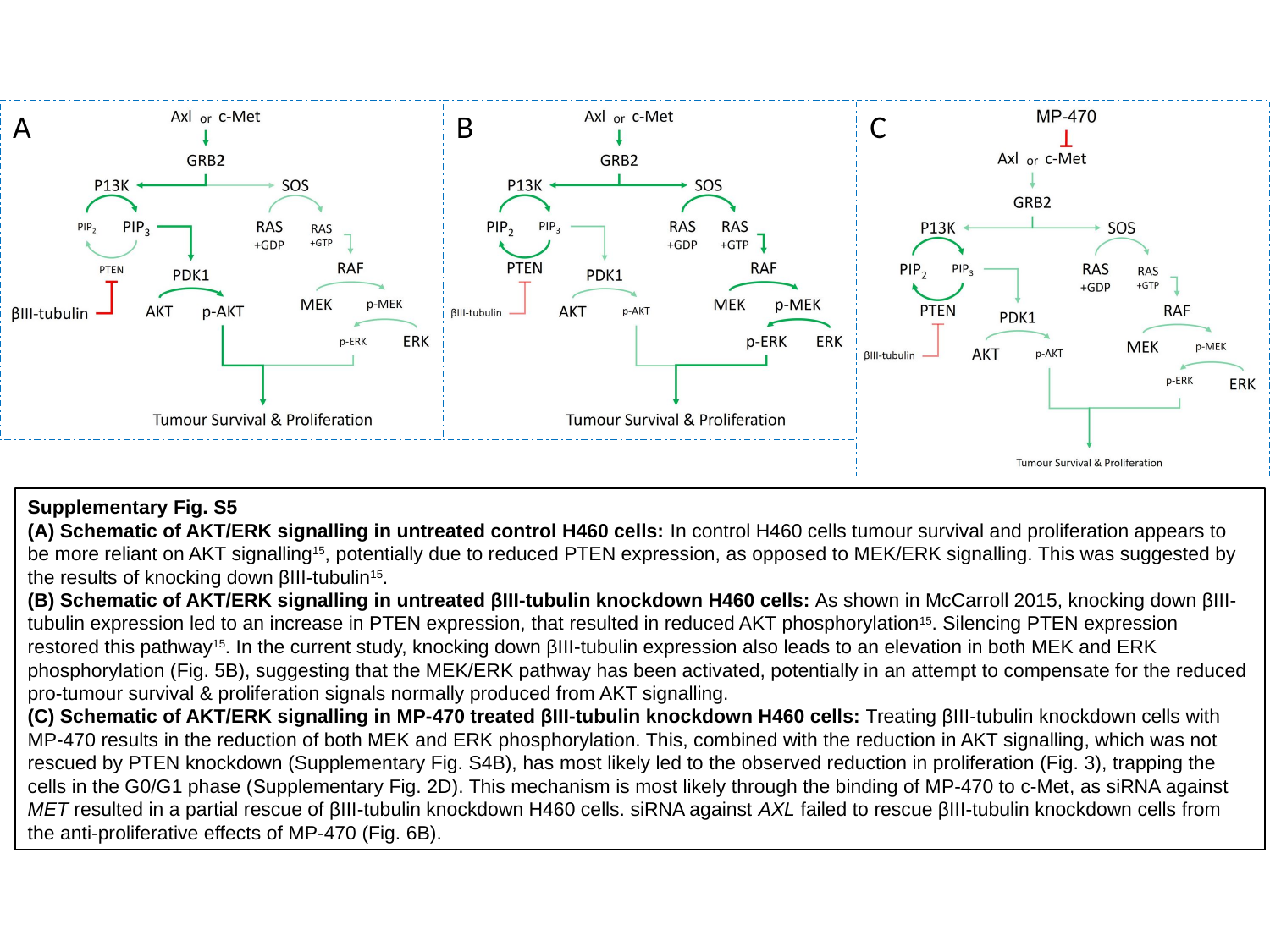

A
B
C
Supplementary Fig. S5
(A) Schematic of AKT/ERK signalling in untreated control H460 cells: In control H460 cells tumour survival and proliferation appears to be more reliant on AKT signalling15, potentially due to reduced PTEN expression, as opposed to MEK/ERK signalling. This was suggested by the results of knocking down βIII-tubulin15.
(B) Schematic of AKT/ERK signalling in untreated βIII-tubulin knockdown H460 cells: As shown in McCarroll 2015, knocking down βIII-tubulin expression led to an increase in PTEN expression, that resulted in reduced AKT phosphorylation15. Silencing PTEN expression restored this pathway15. In the current study, knocking down βIII-tubulin expression also leads to an elevation in both MEK and ERK phosphorylation (Fig. 5B), suggesting that the MEK/ERK pathway has been activated, potentially in an attempt to compensate for the reduced pro-tumour survival & proliferation signals normally produced from AKT signalling.
(C) Schematic of AKT/ERK signalling in MP-470 treated βIII-tubulin knockdown H460 cells: Treating βIII-tubulin knockdown cells with MP-470 results in the reduction of both MEK and ERK phosphorylation. This, combined with the reduction in AKT signalling, which was not rescued by PTEN knockdown (Supplementary Fig. S4B), has most likely led to the observed reduction in proliferation (Fig. 3), trapping the cells in the G0/G1 phase (Supplementary Fig. 2D). This mechanism is most likely through the binding of MP-470 to c-Met, as siRNA against MET resulted in a partial rescue of βIII-tubulin knockdown H460 cells. siRNA against AXL failed to rescue βIII-tubulin knockdown cells from the anti-proliferative effects of MP-470 (Fig. 6B).

## Slide 6
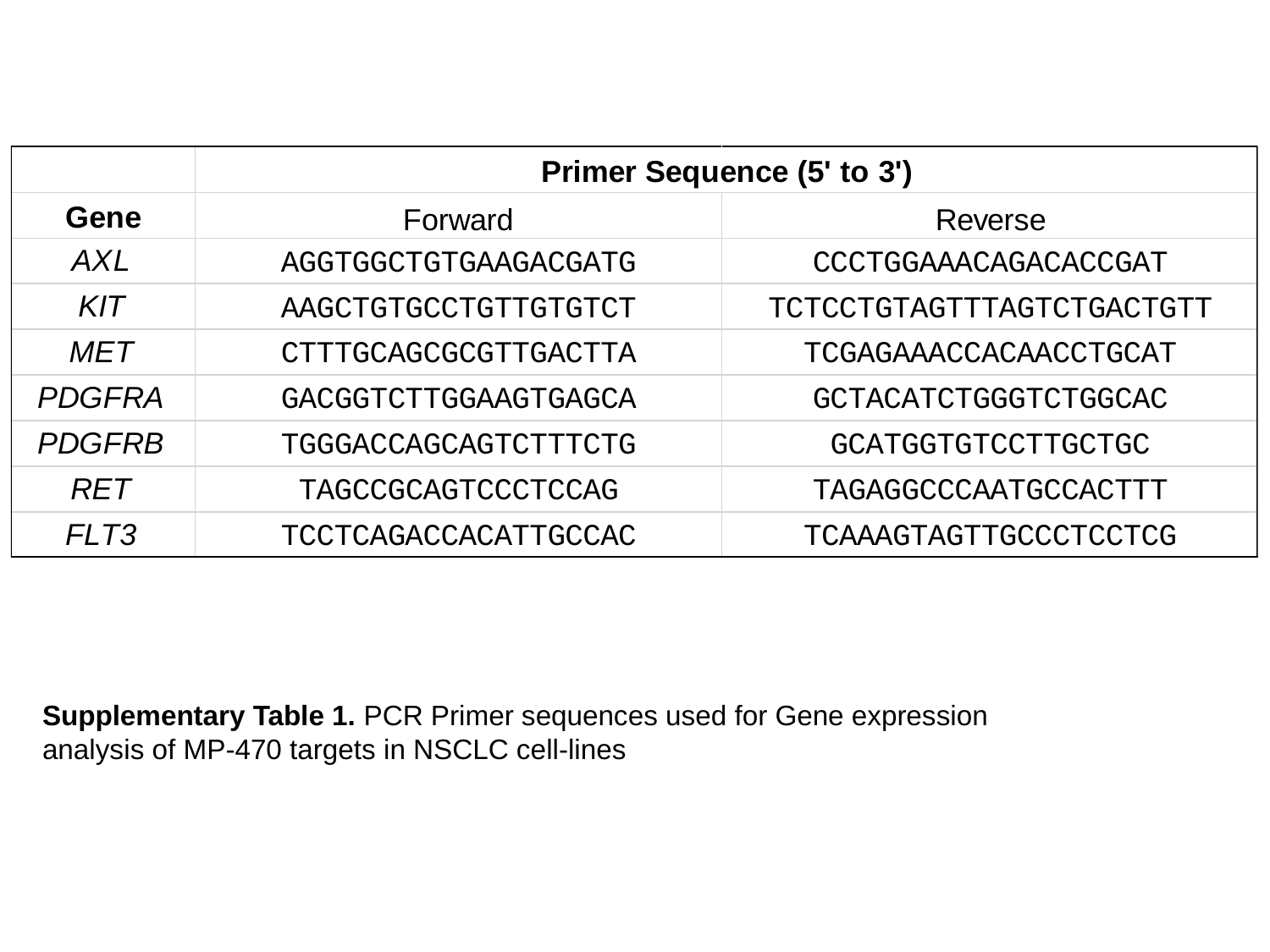

Supplementary Table 1. PCR Primer sequences used for Gene expression analysis of MP-470 targets in NSCLC cell-lines
